# Upcycling Waste PET: I. Ammonolysis Kinetics of Model Dimethyl Terephthalate and the Catalytic Effects of Ethylene Glycol

**DOI:** 10.1021/acssuschemeng.4c10238

**Published:** 2025-03-06

**Authors:** Richard-Joseph L. Peterson, Elanna P. Neppel, Lars Peereboom, P. Anh Trinh, Robert Y. Ofoli, John R. Dorgan

**Affiliations:** Chemical Engineering and Materials Science Department, Michigan State University, East Lansing, Michigan 48823, United States

**Keywords:** plastic waste, poly(ethylene terephthalate), recycling, upcycling, ammonolysis, terephthalamide, reaction kinetics

## Abstract

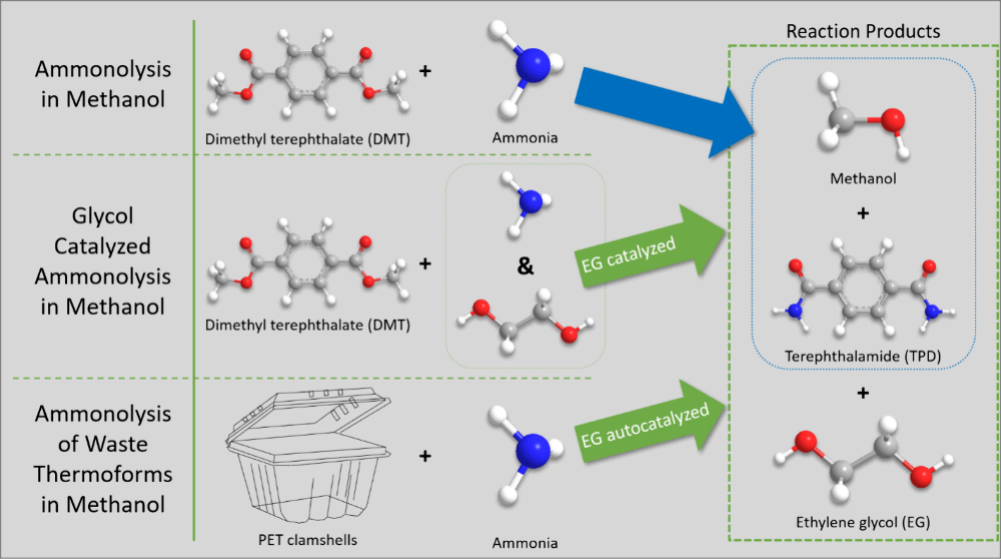

Chemical upcycling of waste plastics can play an important role in developing greater circularity in the material flows associated with the plastics industries. In this study, a fundamental understanding of upcycling poly(ethylene terephthalate) (PET) using ammonolysis is established. First, rate constants are determined for model studies of the ammonolysis of dimethyl terephthalate (DMT) in methanol. Ammonolysis proceeds sequentially, and a first ester group of DMT reacts with ammonia to produce methanol and the monoamide methyl 4-carbamoylbenzoate (MCB). Next, MCB reacts with ammonia to yield methanol and terephthalamide (TPD). At 100 °C, the pseudo first order rate constants are *k*_1_^′^ = 0.25 ± 0.02 h^–1^ and *k*_2_^′^ = 0.11 ± 0.02 h^–1^. Experiments conducted at 50, 75, 100, and 125 °C yield activation energies for the first and second reactions of *E*_*a*1_ = 27.9 ± 2.2 kJ/mol and *E*_*a*2_ = 37.3 ± 3.3 kJ/mol. Ammonolysis is demonstrated to be catalyzed by ethylene glycol (EG) with a first order concentration dependence. At 100 °C with EG present in a 3:1 excess, the pseudo first order rate constants are *k*_3_^′^ = 6.3 ± 0.7 h^–1^ and *k*_4_^′^ = 1.7 ± 0.3 h^–1^, representing a 22-fold increase. Demonstration experiments with reclaimed mixed postconsumer thermoform containers reveal that the ammonolysis of PET is self-catalyzed by the generated EG; the upcycling reaction on polymer substrates is autocatalytic. This new detailed understanding of the self-catalyzed chemical kinetics of ammonolysis suggests EG as the natural choice for the solvent, a topic pursued in part II of this work.

## Abstract 2

Chemical upcycling of PET via ammonolysis may enable economic recovery of landfill-bound PET waste, thus increasing sustainability and promoting a circular economy.

## 1 Introduction

The unique properties of polymeric materials produce both benefits and detriments. Use of lightweight plastic for food packaging saves fuel during transport and reduces food waste due to damage and spoilage.^1−3^ However, many plastics are recalcitrant in terms of natural degradation processes in the environment. Plastics that are improperly disposed of can be released into the environment and, upon weathering, become microplastics. Such microplastics infiltrate the food chain and are now found in human blood and feces.^4−6^ Long-term human health effects are unknown. Additionally, degradation of plastics based on nonrenewable fossil resources produces carbon dioxide and methane emissions that contribute to climate change.^7,8^ The benefits of plastic materials require their continued use, but deleterious effects should be mitigated.

One approach for potentially improving the environmental consequences of plastics use is through developing better recycling technologies. Global production of plastics is estimated as 400 million tons in 2021, so there is an abundance of material available.^9^ However, only 9% of plastics are recycled across the United States.^10^ Reasons that the recycling rate are so low include a low economic incentive for recycling and the fact that plastics irreversibly degrade over multiple mechanical recycling iterations.^11,12^

There are several technological alternatives to mechanical recycling. Chemical recycling, also called molecular recycling, breaks down plastics into new molecular building blocks. In some cases, these building blocks can be used to make virgin grades of the materials from which they are derived. Chemical upcycling refers to creating new materials with greater economic value than the original source polymer.

Although poly(ethylene terephthalate) (PET) is recyclable, most of it ends up being discarded into the environment, incinerated, or dumped in landfills.^13^ PET accounts for 6% of total plastic production, making it the fifth most produced plastic.^10^ But only 30% of waste PET is recycled each year^14^ with the majority being mechanical “bottle-to-bottle” recycling. However, PET is a promising candidate for chemical recycling.^15^

It has been known for a long time that the chemical structure of PET makes it well suited for chemical recycling.^16−21^ There is inherent value in the aromatic moiety found in its backbone and many chemical recycling strategies coproduce EG, an easily marketed commodity chemical. PET backbone linkages are susceptible to attack from various reactive species. The Eastman Chemical Company operates industrial glycolysis^22−25^ and methanolysis^26−36^ facilities for molecular recycling of PET.^37−40^ Other potential processes for chemical recycling include hydrolysis,^41−46^ aminolysis,^47−52^ and ammonolysis. Of these processes, ammonolysis is the least studied.

No detailed studies of the relevant chemical kinetics of PET ammonolysis are available in either the patent or journal literature. It is known that hydrolysis of PET under hydrothermal conditions using a dilute aqueous ammonia solution leads to minimal formation of terephthalamide.^53^ For direct ammonolysis, Zengel and Bergfeld generated terephthalamide (TPD) from both DMT and PET feedstocks.^54−56^ These authors suspend PET in ethylene glycol and add gaseous ammonia.^57^ However, other than simple conversion vs time data at one operating temperature, no chemical kinetics are reported.

The present study determines quantitative rate data that can enable the design of modern chemical upcycling processes for PET. To determine ammonolysis kinetic parameters, model studies using 7N ammonia dissolved in methanol are performed on dimethyl terephthalate (DMT). Experiments are conducted to determine the rate constants over a range of temperatures thus providing the Arrhenius parameters for each reaction. Based on older literature on small molecule esters,^58−60^ the hypothesis that ammonolysis is catalyzed by EG is tested and confirmed. Experiments establish that ammonolysis of DMT in methanol is first order with respect to EG concentration. Recovered PET waste plastic is used to compare observed conversion rates to those obtained in model studies; this comparison demonstrates the autocatalytic nature of the ammonolysis of PET. Finally, the effects of particle size are briefly examined. Typical thicknesses of PET flakes derived from shredding PET thermoforms do not produce significant diffusional limitations. Concluding remarks focus on how the upcycling process can be improved and how it can be easily integrated into the existing infrastructure for chemical recycling of PET by methanolysis and glycolysis.^37−40^

## 2 Materials and Methods

Materials were secured from commercial and noncommercial sources. Waste PET was obtained from the Michigan State University recycling center. DMT, DMSO-d6, and EG were purchased from Sigma-Aldrich. TPD was purchased from Combi-Blocks and nitrogen was purchased from Air-Gas.

Reactions were conducted in a Parr 5000 multireactor system utilizing a Parr 4870 process controller with pressure and temperature data recording. Details are available in the Supporting Information (SI). Briefly, the multireactor system had 6 individual 75 mL stainless steel reactors; each reactor had a Swagelok fitting connection to a pressure manifold, O-ring seals and a screw on caps reinforced with bolts (rated to 5000 psi). The reactors were glass lined, contained a magnetic stir bar, and sat in a heated well with temperature control. Excellent mixing was provided by an integrated magnetic stirrer set to 800 R.P.M.. As described in the S.M., reactions were halted by quenching the entire reactor in ice water.

To conduct ammonolysis of DMT, the as received DMT pellets were crushed into a fine powder using a mortar and pestle. The crushed DMT was dried in a vacuum oven overnight, weighed (1.0 g) and placed into the 75 mL Parr reactors. The reactors were closed and rubber septa were used to cover the Swagelock opening. Using a syringe, 15 mL of 7N ammonia in methanol solution was injected through the rubber septa into the reactors. The reactors were placed in their heating mantles, the septa were removed, and a nitrogen gas line was connected. The reactors were charged with 40 bar of ultrahigh purity (99.999%) nitrogen. The stir rate was set to 800 rpm.

Reactions were halted after a specified reaction time. The reactor was removed from the heating mantle and quenched by submersion in an ice bath until it reached 5 °C. The reactor was then vented, opened, and the glass insert was removed. A 1 M hydrochloric acid solution (10 mL) was immediately added, the mixture was stirred for 15 min, and the crystallized products were recovered via vacuum filtration. The recovered solids were dried in a convection oven at 60 °C.

Products were analyzed using nuclear magnetic resonance (NMR) spectroscopy (Bruker 500 MHz). Products were dissolved in DMSO-d6 and proton spectra were generated using a 2-s relaxation delay and 32 scans. Peak integrations were performed using the software MestreNova from Mestrelab.

Catalytic effects were investigated by adding ethylene glycol to the reaction mixture and following the procedures described previously. The molar ratio of EG to DMT varied from 1, to 2, to 3 by changing the amount of glycol added to the ammonia solutions.

Postconsumer, thermoform PET containers were collected from the MSU recycling center, hand washed with mild dish soap, well rinsed, and cut into approximately 1 cm × 1 cm flakes. Some of this flake material was ground in a commercial blender (Waring model CB15) and separated using a series of sieving trays mounted on a motorized agitation tray (Humbolt Industries). The material fraction having particle sizes between 150 and 250 μm was collected for use in experiments.

## 3 Results and Discussion

### 3.1 Thermochemistry

**Scheme 1:**
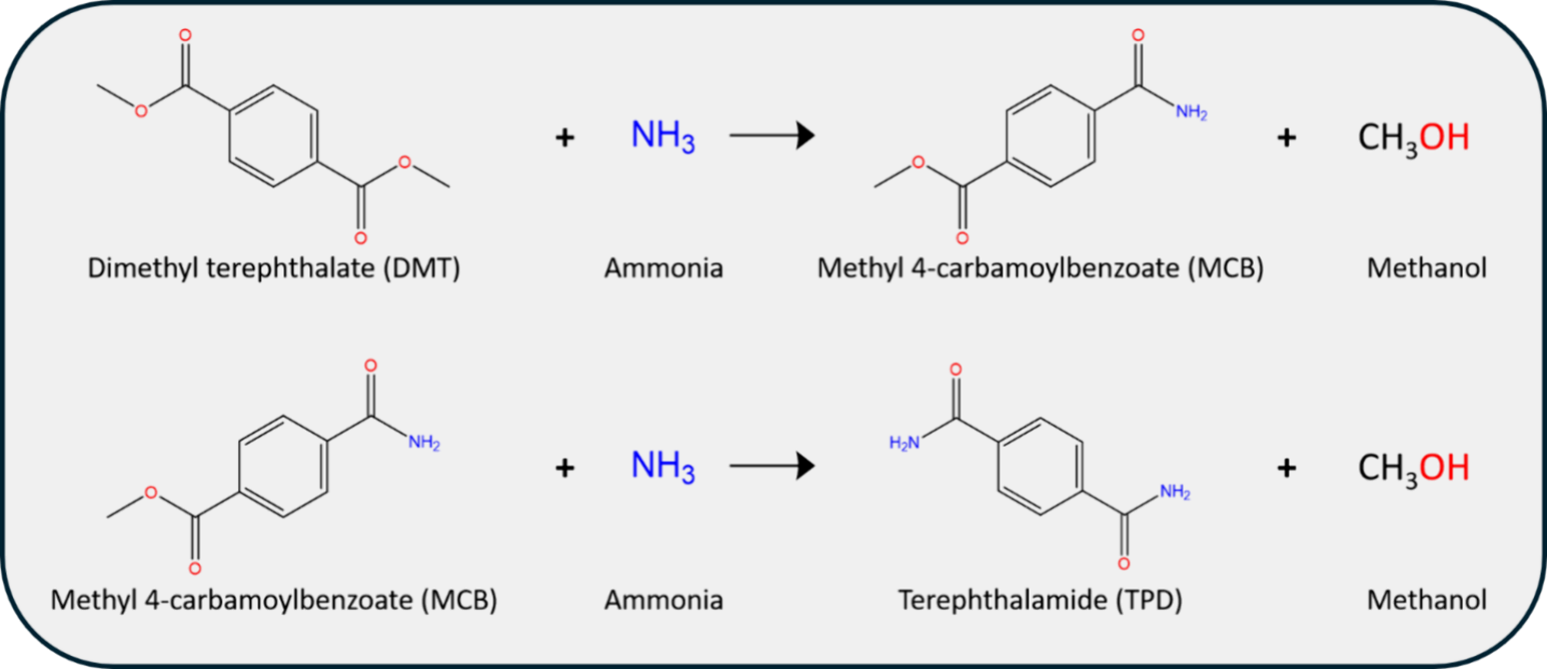
Reaction Scheme for Dimethyl Terephthalamide (DMT) to Terephthalamide (TPD) Ammonia attacks one of the two equivalent ester bonds of DMT to give the monoamide, methyl(4-carbamoylbenzoate) (MCB). Ammonia reacts with the second ester of MCB to yield the TPD. Each step consumes one ammonia and produces one methanol.

Before discussing chemical kinetics, it is prudent to examine the underlying thermochemistry of the reactions shown in Scheme 1. The equilibrium constant, *K,* is related to the Gibbs free energy of reaction, Δ*G*_*rxn*_, through the elementary relationship of eq 1,
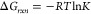
1where R is the ideal gas constant and T is the absolute temperature. In a first approximation, the entropy of reaction may be ignored and the enthalpy of reaction, Δ*H*_*rxn*_, used in place of the free energy. Enthalpy of reaction can be estimated as, “the sum of the bonds broken minus the sum of the bonds formed”. For the reaction of DMT to MCB, using bond values from LibreTexts^61^ gives a value of Δ*H*_*rxn*_ = −23 *kJ*/*mol*. Since the same number and kinds of bonds are broken and formed, the corresponding value for the conversion of MCB to TPD is also Δ*H*_*rxn*_ = −23 *kJ*/*mol*. These first estimates indicate exothermic reactions, which are usually easily performed. To obtain a more accurate estimation, neighboring electronic effects can be captured using group contribution methods; using the procedure described in the SM based on L. Constantinou’s work,^62^ gives a Gibbs free energy of Δ*G*_*rxn*_=-10.54 kJ/mol for each reaction step. These revised values correspond to an equilibrium constant of K_1_ = 30.0. Clearly, these estimated values indicate the reactions are thermodynamically feasible in high yields and justify an exploration of their kinetics.

Because of its critical role, careful attention was paid to accurately determining the average temperature experienced over the course of the reaction. Nominal temperatures are presented in Table 1 and correspond to the set point temperature for the feedback controller of the Parr reactor system. PID feedback control produces dynamic temperature profiles. The temperature takes about 30 min to equilibrate; typical temperature profiles are shown in the SI. Factors that impact the profile include set-point temperature and the specific reactor and thermal well used. Average temperatures throughout each run were determined by the mean value theorem. Like temperature, accurate determination of the composition of the reaction mixture as a function of time is very important.

**Table 1:** Reactions Times Used for Each Set Point Temperature

set point temperature (°C)	reaction durations (hours)
50	2, 6, 24, 48, 72, 120, 150, 168
75	3, 6, 18, 24, 30, 48, 96
100	3, 5, 9, 13, 22, 48, 96
125	1, 3, 4.5, 7, 18, 24, 48

### 3.2 Compositional Analysis

**Figure 1:**
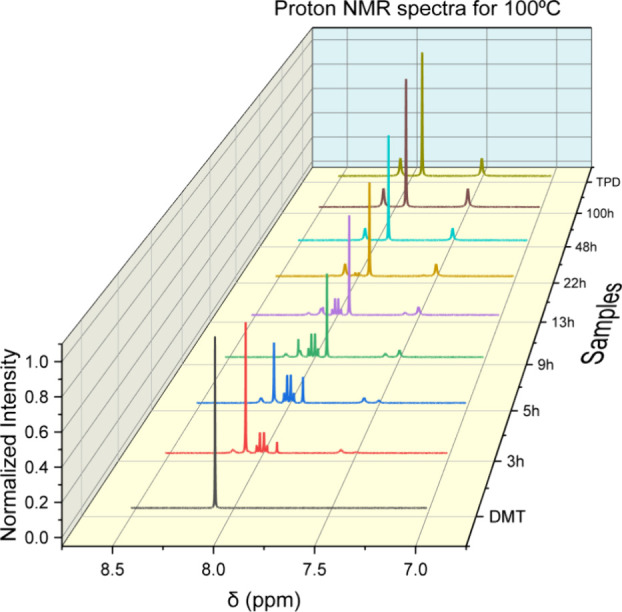
Proton NMR spectra for DMT, TPD, and a representative sample that ran at 100 °C for 100 h. Each of the compounds of interest has 4 protons in the aromatic region. Peak assignments are Terephthalamide 1H NMR ((500 MHz, DMSO) δ 8.06 (s, 2H), 7.91 (s, 4H), 7.49 (s, 2H)), Dimethyl Terephthalate 1H NMR ((500 MHz, DMSO) δ 8.06 (s, 4H), 3.87 (s, 6H)), and Methyl 4 carbamoylbenzoate (6757–31–9) 1H NMR ((500 MHz, DMSO) δ 8.15 (s, 1H), 8.03–7.93 (m, 4H), 7.56 (s, 1H), 3.85 (s, 3H)).

It is necessary to assign peaks in proton NMR spectra to a specific compound before determining mole fractions. Figure 1 shows peak assignments for DMT and TPD based on spectra of recrystallized standards. Also shown are spectra for samples recovered at different reaction times at 100 °C. No MCB standard was available, but peak assignments became obvious. Over the course of the reaction, as the NMR peaks related to DMT diminish, unknown peaks grow and as the unknown peaks are extinguished, the peaks related to TPD grow. The unknown peaks consist of a multiplet between 8.03 and 7.93 ppm that integrates to four, two broad peaks at 8.15 and 7.56 ppm that integrate to one each, an ester peak at 3.85 ppm that integrates to three. Based on the location of the peaks, the integrations, and the context of the reactions, the “unknown” peaks are assigned to the reaction intermediate, MCB. The peak assignments are thus as follows: Terephthalamide ^1^H NMR ((500 MHz, DMSO) δ 8.06 (s, 2H), 7.91 (s, 4H), 7.49 (s, 2H)), Dimethyl Terephthalate ^1^H NMR ((500 MHz, DMSO) δ 8.06 (s, 4H), 3.87 (s, 6H)), and Methyl 4 carbamoylbenzoate (6757–31–9) ^1^H NMR ((500 MHz, DMSO) δ 8.15 (s, 1H), 8.03–7.93 (m, 4H), 7.56 (s, 1H), 3.85 (s, 3H)). With peaks assigned, mole fractions of the recovered solids are calculated from the relative ratios of the peaks as described in the SI.

To ensure accurate compositional measurement, mole fractions of each species are determined using proton NMR spectroscopy. Mole fractions are calculated by integrating the peaks in the aromatic region and determining the relative ratio of each species to one another. An in-depth explanation and an example calculation can be found in the SI. Having carefully determined the composition of the reacting mixtures as a function of time and temperature, these primary data can be analyzed in the context of a conventional chemical kinetics model.

The first step in determining what type of kinetic model should be used is to examine the nonideality of the reaction mixture. To examine nonidealities, the software package ASPEN Plus^63^ is used to calculate activity coefficients in the liquid phase. Several different well-established thermodynamic models, namely PC-SAFT,^64,65^ NRTL,^66−68^ and UniQUAC^69^ are used. The calculated activity coefficients were 5–15% away from unity. Details of the activity calculations are provided in the SI. Application of activity corrections when regressing the primary data to the kinetics model did not improve the goodness of fit metrics. To facilitate the widest possible use of the results, mole fractions are adopted as the primary dependent variable in the chemical kinetics model.

### 3.3 Chemical Kinetics Model Development

The chemical kinetics model is developed in a conventional fashion and simplified using several well justified assumptions. The rate at which ammonia is consumed is expressed as,
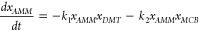
2where *x*_*AMM*_, *x*_*DMT*_, and *x*_*MCB*_ are the mole fractions of ammonia, DMT, and MCB, respectively. The rate constant *k*_1_ governs the first conversion of an ester to an amide and *k*_2_ is the rate constant of the second ester conversion. Here and elsewhere the reactions are considered irreversible based on the large equilibrium constants estimated based on the thermochemistry calculations. The model developed fits the experimental data very well providing further justification for treating the reactions as irreversible.

Due to the large excess, the ammonia concentration changes very little and is therefore considered constant. This allows the concentration of ammonia to be combined with the fundamental rate constant giving eqs 3-5.
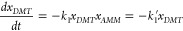
3
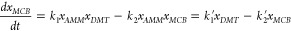
4
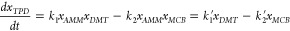
5

In eqs 3-5*x*_*TPD*_ represents the mole fraction of TPD and *k*_1_^′^ and *k*_2_^′^ are the pseudo first order rate constants for reaction step one and step two, respectively.

Differential eqs 3-5 can be solved analytically using Laplace transforms resulting in eqs 6-8 (details appear in the SI).
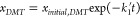
6
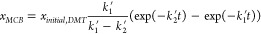
7
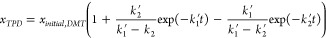
8

In eqs 6-8, t represents time and *x*_*initial*, *DMT*_ is the mole fraction of DMT at time t = 0.

Equations giving the mole fraction of each species as a function of time *and temperature* are desirable and may be obtained through application of the Arrhenius equation. Each of the rate constants is governed by its own pre-exponential (“collision factor”) and activation energy as expressed in eqs 9 and 10
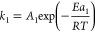
9
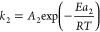
10Here, A_1_, A_2_, Ea_1_, and Ea_2_ are the corresponding collision factors and activation energies of the two reactions.

Activation energies and pre-exponential factors are determined by regressing model parameters in eqs 6 to 10 against the experimental data, which consists of mole fractions, temperatures, and reaction times. The average temperature was measured over the total reaction time and this average was designated as the assigned reaction temperature for that specific data set. Using the Python programming language, the library curve_fit in scipy.optimize is used to perform a fit of the data to the model and return the activation energies and pre-exponential factors as fitting parameters. For each reaction, the solver is fed an array of the mole fractions, average temperatures, and associated reaction times. The root mean squared error is minimized so that by fitting the model to the reaction data, the “best fit” activation energies and pre-exponential factors are returned. Example code is provided in the SI.

**Figure 2:**
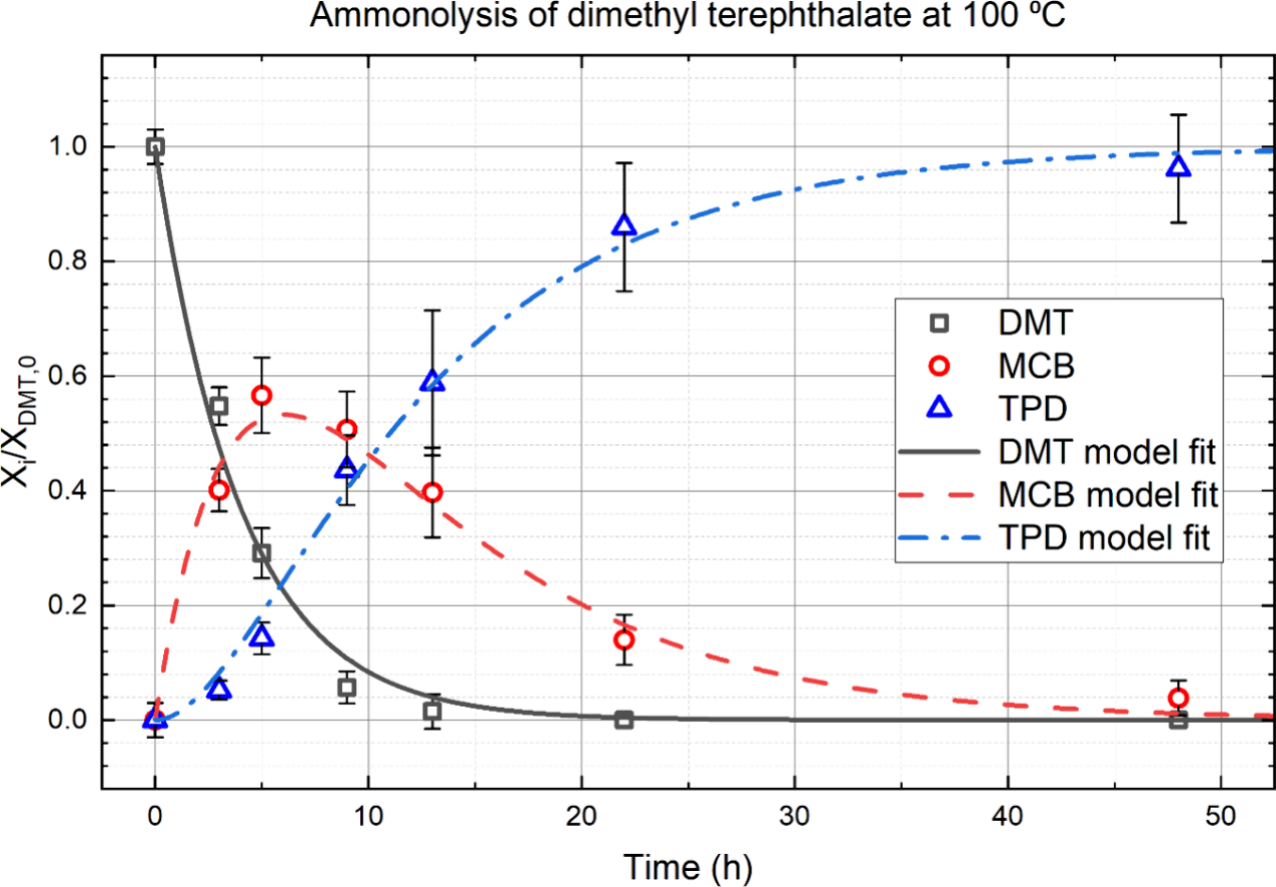
Measured mole fractions of each component (points) and model fit (lines) as a function of time. The intermediate, MCB, passes through a maximum of about 60 mol % at 5 h. The product, TPD, is aches full conversion after 48 h with yields of 89–96%.

The adopted model does an excellent job of fitting the data with R^2^ values of 0.997,0.987, and 0.995 for curves “A”, “B” and “C” respectively. Figure 2 shows measured mole fractions (points) and model fits (lines) as a function of time for a nominal reaction temperature of 100 °C (see SM for 50 °C, 75 °C, and 125 °C). The reactant, DMT, is consumed as time increases. The intermediate, MCB, is generated, achieves a maximum concentration, and is ultimately consumed. The final product, TPD, is generated slowly at first, then more rapidly, and achieves its final concentration by 48 h. Conversion is excellent; the yield of TPD ranged from 89 to 96 mol % of the theoretical amount.

**Figure 3:**
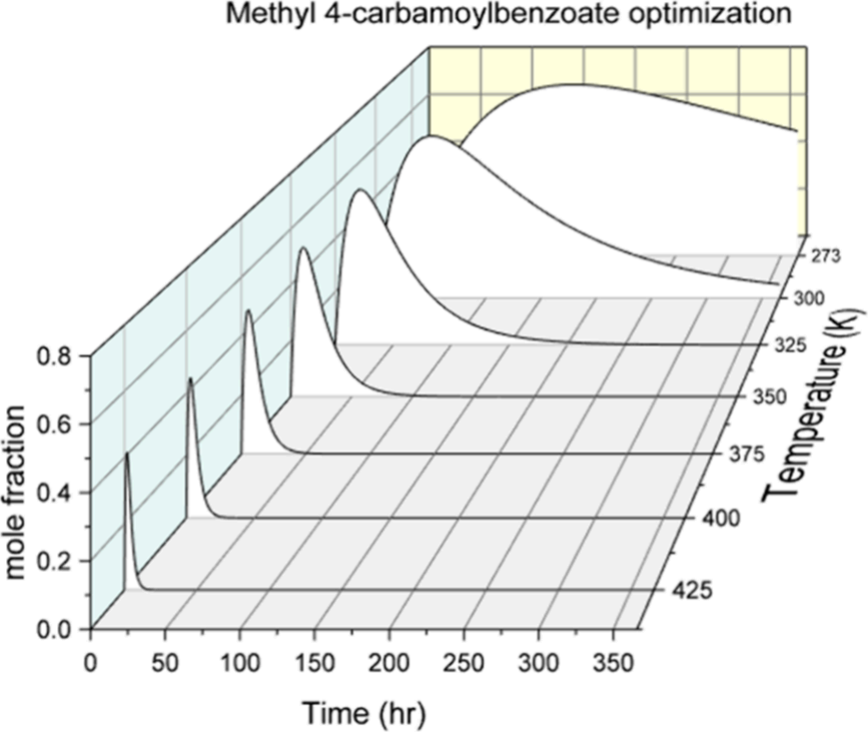
Calculated MCB mole fractions versus time for temperatures from 273 to 425 K. The peak mole fraction of MCB ranges from 0.40 to 0.72 and the peak broadens significantly at low temperatures. It is possible to target the generation and isolation of MCB which can be used in alternative upcycling cascades.

An interesting aspect of the system is that the reaction intermediate, MCB, passes through a large and broad maximum, peaking at about 60 mol %. Figure 3 shows model predictions of the mole fraction of MCB versus time for temperatures ranging from 273 to 425 K. The maximum concentration of MCB increases as reaction temperature decreases. The peak also broadens with decreasing temperature. Overall, this behavior indicates the MCB intermediate can be easily isolated as a reaction product.^70^

The activation energies of the ammonolysis of DMT are found to be 27.9 ± 2.2 kJ/mol and 37.3 ± 3.3 kJ/mol; these values are consistent with previously reported data on related systems. Wetzel et al.^71^ performed ammonolysis on various acetates including methyl, ethyl, n-propyl, and isopropyl, finding activation energies that range from 24.8 to 33.8 kJ/mol. The ammonolysis of the most simple aromatic ester, methyl benzoate was studied^72^ and estimated to have an activation energy of 96 kJ/mol^73^ but this value falls to 39.3 kJ/mol when catalyzed by clay.^74^ Gordon et al.^60^ state that primary alcohols serve as catalysts for ammonolysis and determine catalyzed activation energies for the ammonolysis of several other esters; determined values range from 39.3 to 53.1 kJ/mol. Catalytic effects of methanol evidently contribute to the lower activation energies found in the present study compared to the values reported by Wetzel. It follows that in interpreting the pseudo-first order rate constants the concentration of methanol might correctly be included in their definitions.

The activation energy determined for the nucleophilic attack of ammonia on the first ester bond of DMT is different than the activation energy for the attack of ammonia on the MCB intermediate. This is true despite the same functional groups being involved in both reactions. Breaking down this two-step reaction into what might be considered conventional functional groups (aromatic methyl ester) would indicate that both steps of the reaction are identical. A methyl ester is attacked by ammonia and replaced by an amide, consuming the ammonia and generating methanol. If the two reactions were equivalent, the activation energy required for the first reaction would be, within the precision of the measurements, identical to that of the second. This simply is not the case.

**Figure 4:**
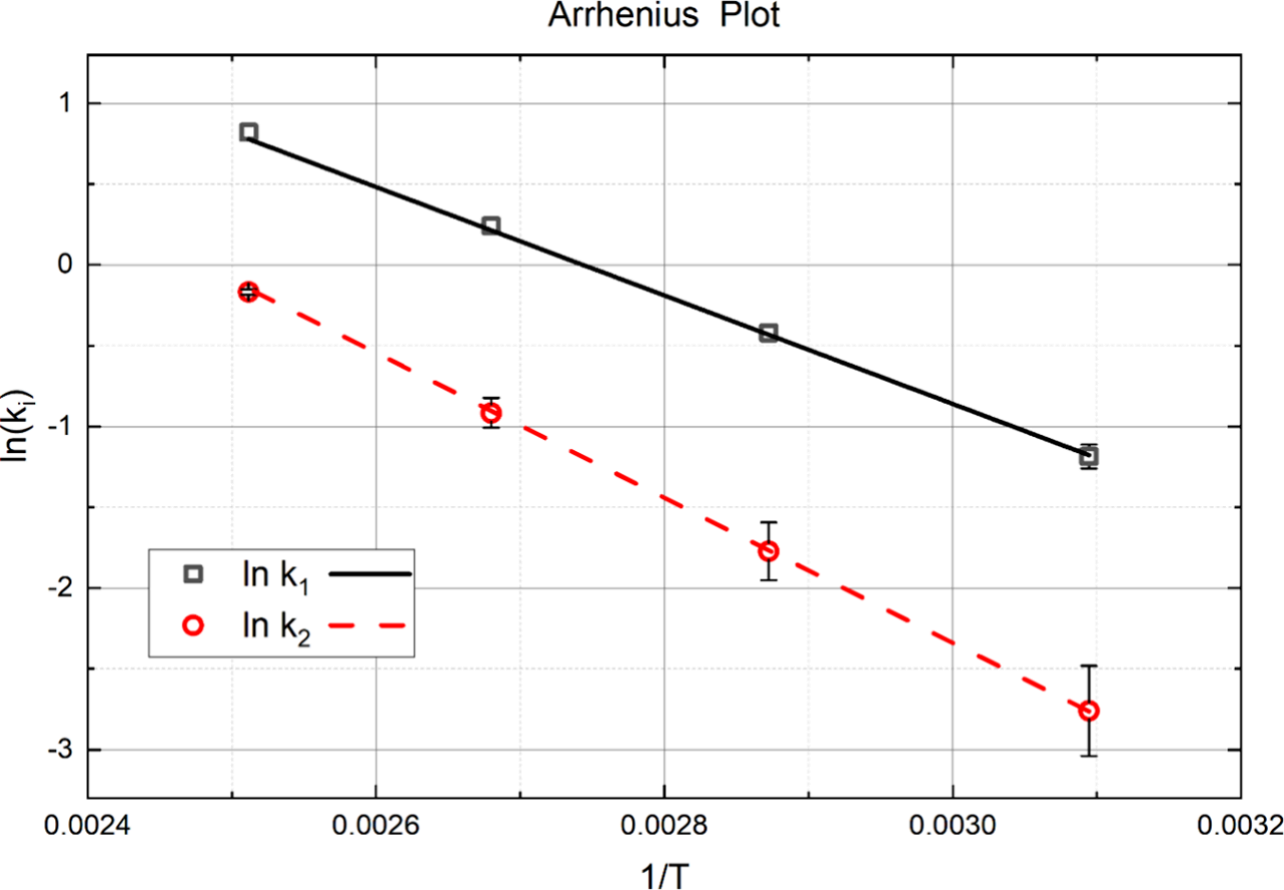
Arrhenius plot according to the reaction data collected.

Figure 4 shows the Arrhenius plot of the two reaction steps for which the ester is converted to an amide. In Figure 4, the y-intercept is related to the pre-exponential factor, *A*, and the slope is related to the activation energy (*Ea*) for each reaction. The activation energy of the first reaction is Ea = 27.9 ± 2.2 kJ/mol, with a corresponding pre-exponential factor of 10031 ± 790 h^–1^. The activation energy of the second reaction is 37.3 ± 3.3 kJ/mol, with a pre-exponential factor of 68529 ± 6056 h^–1^. The slope of ln(*k*_2_) is steeper than that of ln(*k*_1_), indicating the difference of activation energy. Clearly, despite being the same reaction based on functional groups, the second step has a significantly higher activation energy.

Collision factor A_2_ is nearly 7 times greater than A_1_. This result implies there are more collisions of ammonia with MCB per unit time compared to DMT. Factors that contribute to increased collision frequencies include diffusivity and molecular size (reaction cross-section). For solute–solvent reactions the collision factor is directly proportional to the diffusivity of the solute, which is a function of molecular weight. In the first reaction step, the reactant undergoes a change in molecular weight from 194.19 g/mol to 179.18 g/mol. This 8% decrease affects diffusivity and therefore the collision rate. An order of magnitude approximation of how the decrease in molecular weight changes the diffusion rate can be obtained using Graham’s Law^75,76^ as expressed by eq 11.
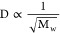
11

In eq 11, D is the diffusivity, and Mw is the molecular weight of the solute. The relative change in diffusivity based on the change in molecular weight is calculated in eq 12.
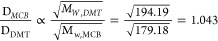
12Where D*_MCB_*, and D_DMT_ are the diffusivities of MCB and DMT and *M*_*W*, *MCB*_ and *M*_*W*, *DMT*_ are the molecular weights of MCB and DMT respectively. The result shows a modest 4.3% increase in diffusivity.

The molecular cross-section (σ_AB_) can be thought of as the average distance between species when they collide and is directly proportional to the collision frequency. The decrease in molecular weight implies a decreased molecular cross-section which would mean a decreased collisional frequency. So, this effect cannot explain the observed differences.

**Figure 5:**
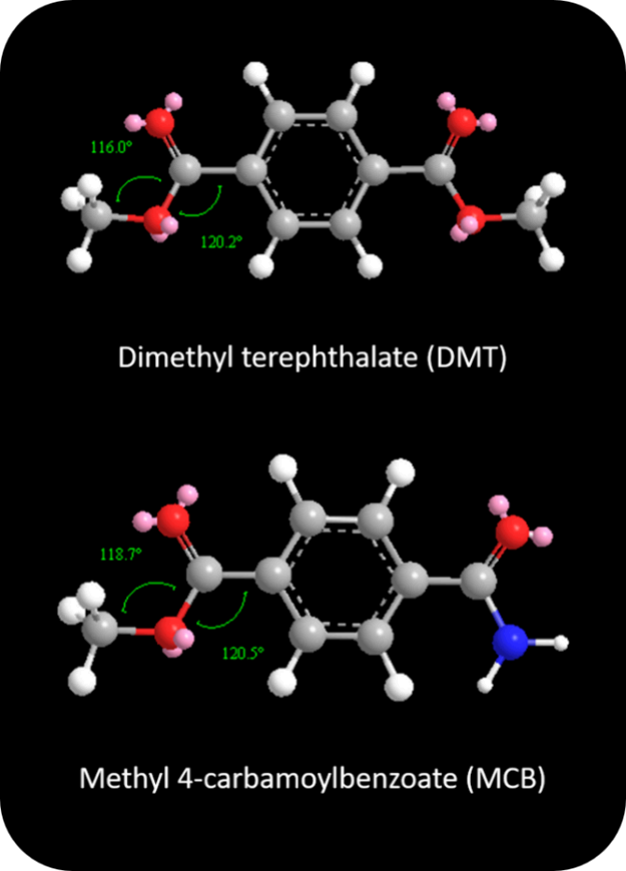
Bond angles near the ester bond for DMT and MCB.

Instead, the higher value of A_2_ compared to A_1_ may be partially explained by the fact that the bond angle of the ester in MCB is greater than the bond angle of the esters in DMT. Bond angles were calculated by using molecular dynamics in the ChemDraw 3D software package to minimize conformational energies. Minimized structures are shown in Figure 5; the ester bond angles are calculated to be 116.0° and 118.7° for DMT and MCB, respectively. Since the pre-exponential is a measure for the number of collisions in the right orientation for reaction, it is reasonable to hypothesize that a more accessible bond orientation, such as a larger bond angle results in a higher collision frequency of properly oriented ammonia molecules. While this explanation is consistent with the observations, it is hard to envision it explaining the entirety of the observed difference.

The activation energy differences between ammonia reacting with DMT versus MCB can be attributed to subtle differences in electronic structure. The ester in MCB has a higher electron density than the esters of DMT. This electronic effect is because the amide group is less electron withdrawing than an ester group. Accordingly, the relatively delocalized electrons of the aromatic ring are more available to the sole ester group in MCB. The availability of this additional electron density renders the carbon of the ester *less* susceptible to nucleophilic attack. This mechanistic reasoning is consistent with the experimentally observed slower rate of reaction and a greater activation energy.

In summary, the data fitting to the model captures all salient experimental observations and provides a useful framework for making predictions over a range of relevant temperatures. However, the liquid medium makes interpretation of the collision factors in classical terms, typically associated with gases, convoluted. Furthermore, the observed difference in activation energies implies that as the temperature is raised, the second reaction would become faster than the first. Such relative reaction rates are implausible. Calculation of the hypothetical crossover temperature where *k*_1_^′^ = *k*_2_^′^ produces a value of 315.0 °C. Such a high temperature is outside the range of validity for the model–at temperatures exceeding about 150 °C it is observed that ammonia reacts with methanol to produce a mixture of methylamine, dimethylamine, and trimethylamine.^77^ The appropriate temperature range for the model is estimated to be from approximately 0–150 °C.

### 3.4 Catalytic Effects of Ethylene Glycol

EG and other glycols have long been known to catalyze the reactions of esters,^58−60^ and such effects must be understood for optimized chemical recycling of PET. Unfortunately, although the catalytic effects of glycols have been known *since 1948*, the corresponding autocatalytic effect is ignored in the literature on PET methanolysis^26−36^ and hydrolysis.^41−46^ To avoid this obvious oversight in the present study, the effects of EG addition are investigated. Based on established literature, the *in situ* production of EG during conversion of PET to TPD should be expected to have an autocatalytic effect.

**Figure 6:**
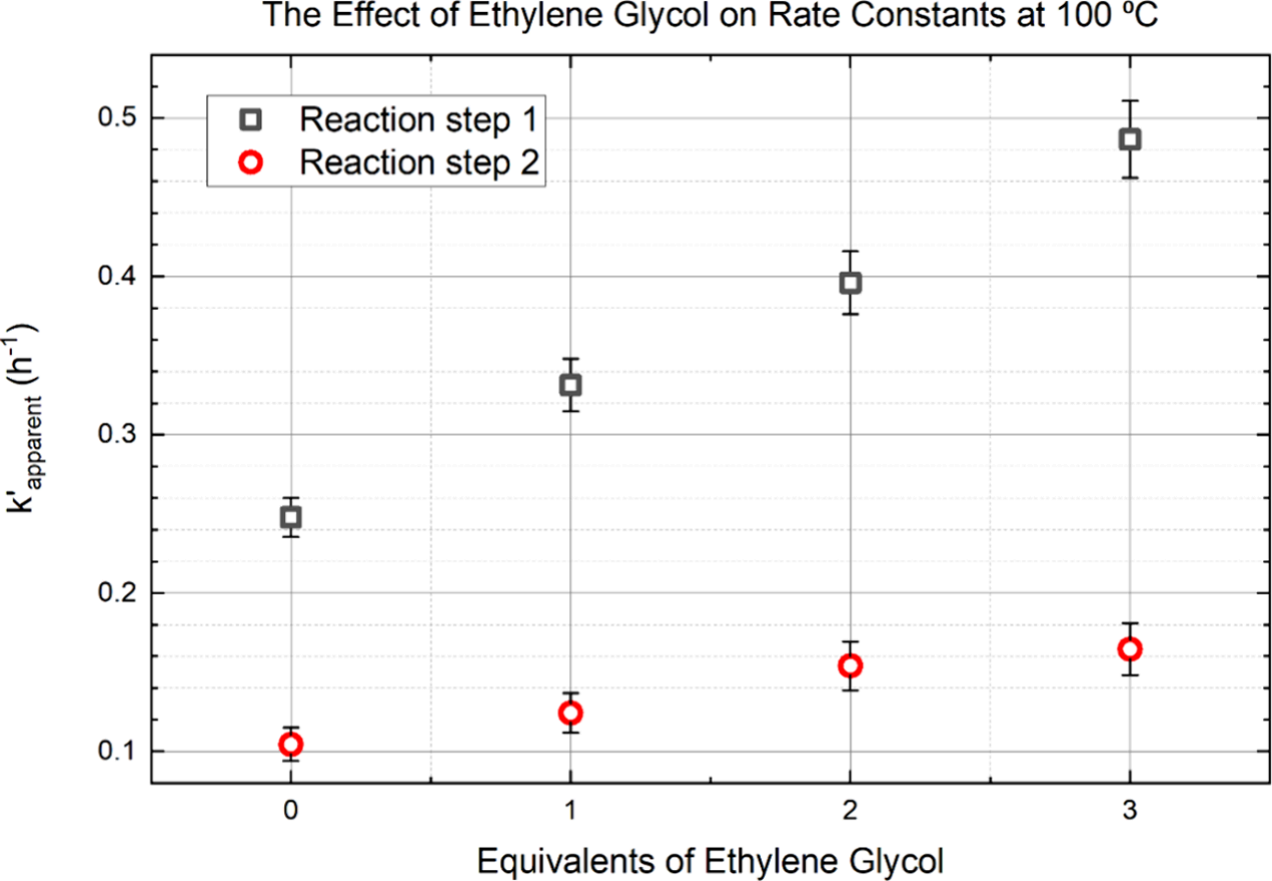
Effect of ethylene glycol on the rate of reaction. Ethylene glycol acts as a catalyst for both reaction steps of the ammonolysis of DMT to TPD.

Figure 6 shows a first order relationship between the reaction rate and the molar ratio of EG-to-DMT. To be clear, Figure 6 shows apparent rate constants, determined by fitting eqs 6-8 to the catalyzed reaction data. This approach lumps the catalytic effect into the pseudo first order rate constants. The observed rate enhancement corroborates the catalytic effect of EG on ammonolysis. The linear nature of the relationship between apparent rate constants and the concentration of EG implies that both reactions are first order with respect to EG. If the rate dependence on concentration were second order, the data would follow a parabolic shape. This observation allows the construction of a new mathematical description.

In the presence of significant quantities of EG, there is a need to derive a set of differential equations that enables a quantitative understanding of the catalytic effect of EG on the ammonolysis reactions. The resulting set of differential equations are presented in eqs 13-16.
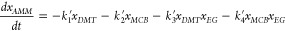
13
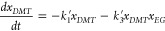
14
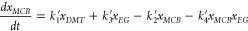
15
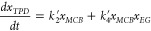
16

Here *x*_*EG*_ is the mole fraction of ethylene glycol, *k*_3_^′^ is the catalyzed pseudo-first order rate constant for the first reaction, and *k*_4_^′^ is the catalyzed pseudo first rate constant for the second reaction. The differential equations can again be solved analytically using Laplace transforms (see SI) giving the results of eqs 17-22.
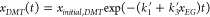
17
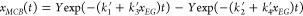
18
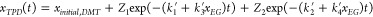
19

In eqs 17-22, auxiliary variables are defined by eqs 20-22.
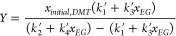
20
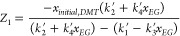
21
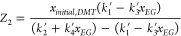
22

**Figure 7:**
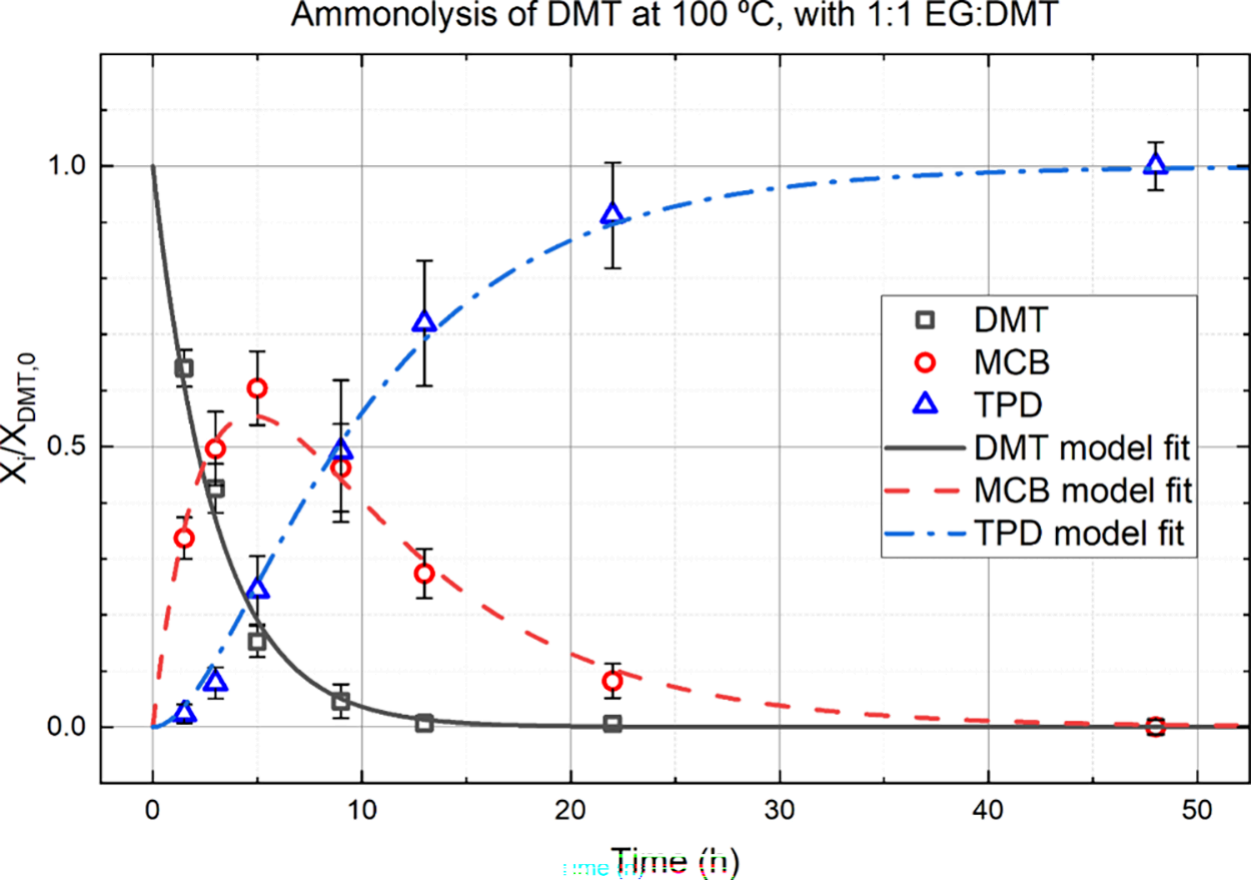
Ammonolysis of DMT in methanol at 100C with 1-to-1 molar ratio of catalytic EG-to-DMT.

The model incorporating catalytic effects is fit to the corresponding data. To do so, the two previously determined rate constants are fixed as *k*_1_^′^ = 0.25 ± 0.02*h*^–1^ and *k*_2_^′^ = 0.11 ± 0.02*h*^–1^. The newly introduced rate constants, *k*_3_^′^ and *k*_4_^′^, are determined as fitting parameters. Figure 7 shows the kinetic data (mole fraction vs time) regressed against the model (fits for the other molar ratios are provided in the SI). The resulting “goodness of fit” (R^2^) values are 0.996, 0.989, and 0.997 for curves A, B, and C, respectively. The new model is fit to the catalyzed reaction data at 100 °C for each EG to calculate the rate constants which are determined to be *k*_3_^′^ = 6.31 ± 0.70 *h*^–1^ and *k*_4_^′^ = 1.65 ± 0.28 *h*^–1^. Rate constants for the catalyzed reactions increased by a factor of 22 times for the first reaction step and 15 times for the second reaction step. Accordingly, EG has considerable catalytic effects.

Figure 7 shows that the overall conversion vs time does not increase dramatically when compared with the results of Figure 2. This is because the concentration of EG is relatively low. In comparison to no EG present, the overall rate of conversion to TPD increases by a factor of 1.6 at a 3-to-1 ratio of EG-to-DMT. Previous studies,^58−60^ show saturation of the catalytic effect at a 3-to-1 molar ratio for monoesters; assuming the ratio of EG to ester groups governs the catalysis, the present catalytic effect should continue up to a 6-to-1 molar ratio for the diester of the present study.

**Figure 8:**
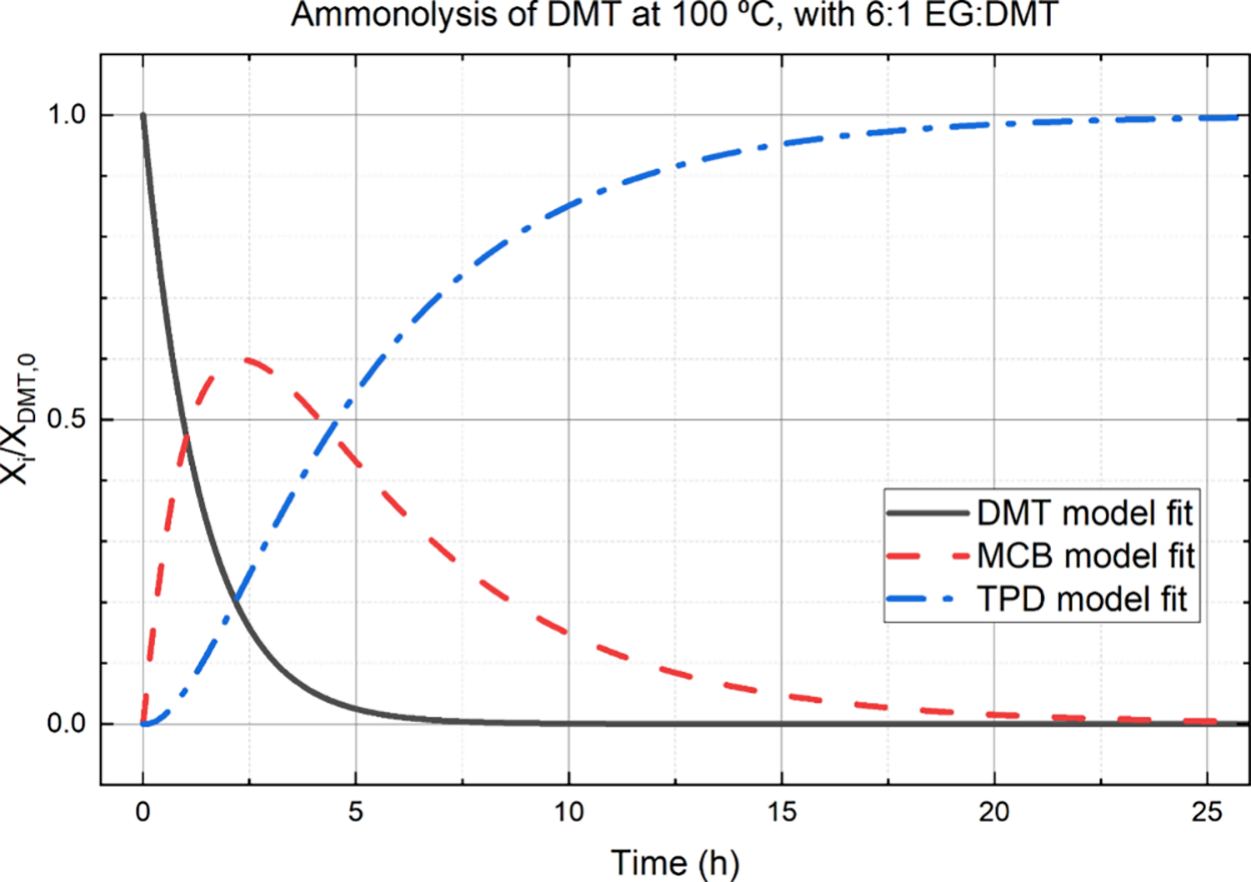
Model predictions for the ammonolysis of DMT at 100 °C using a 6-to-1 molar ratio of EG-to-DMT.

Under the assumption that it is the ratio of EG to ester bonds that governs the catalysis, predictions based on the model for a 6-to-1 molar ratio are presented in Figure 8. The graph demonstrates the reaction should be completed in about 20 h. This represents a 2.3 times decrease in the time required for complete conversion compared to having no added EG present (compare Figure 8 to Figure 2). The rate increase illustrates the importance of the catalytic effect of EG on the ammonolysis of esters. With this better understanding of the catalytic effects of EG, ammonolysis of polymer substrates can be conducted and understood.

### 3.5 Ammonolysis of Waste PET Thermoforms

**Scheme 2:**
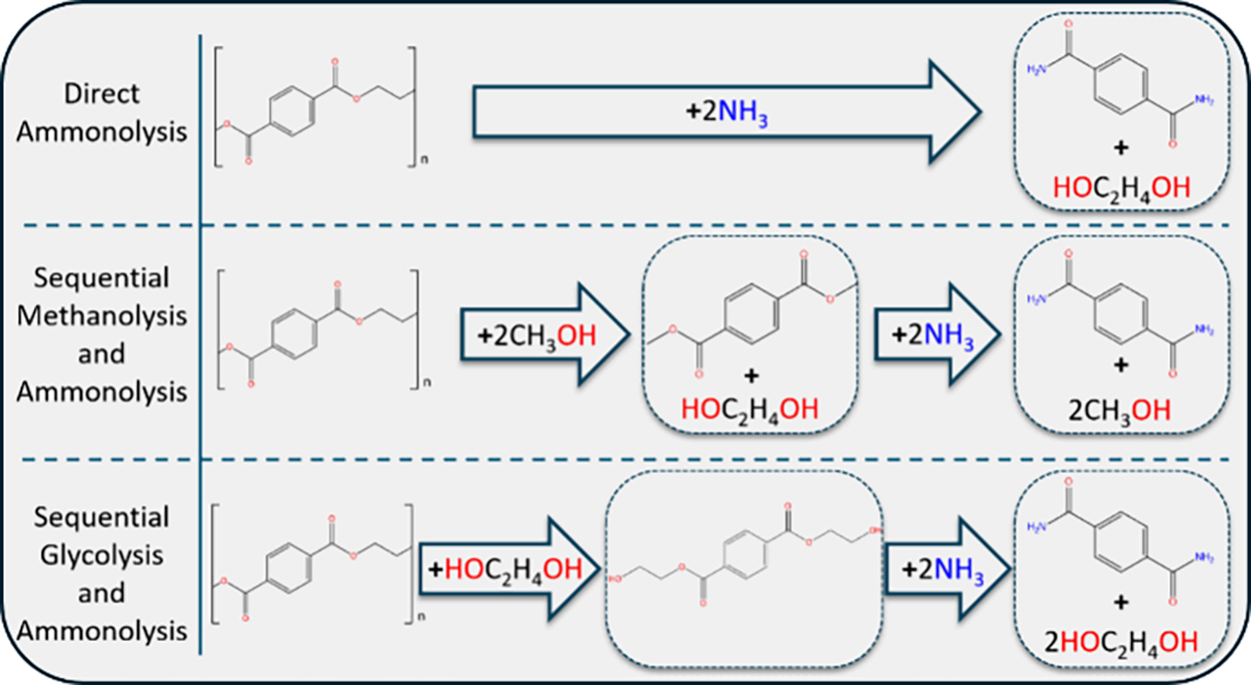
Reaction Scheme for the Depolymerization of PET including Direct Ammonolsys, Sequential Methanolysis and Ammonolysis, and Sequential Glycolsyis and Ammonolysis The reaction pathways combine in the ammonolysis of PET conducted in this work.

As illustrated in Scheme 2, the ammonolysis of PET is considerably more complicated than the ammonolysis of DMT. Ammonia, methanol and *in situ* generated EG each play a role in depolymerization. Furthermore, if the size of the PET solids used is large, significant diffusional limitations may be present. Reaction with PET is similar to DMT because both involve cleaving an ester bond to form an amide. However, since both methanol and ammonia are present, there are multiple reaction pathways to TPD. Significantly, PET transesterification with methanol converts the ester group in the repeating chain to a molecularly distinct methyl ester. In contrast, transesterification of DMT with methanol simply yields DMT. For the polymer case, ammonolysis can occur at two distinct sites, the ester of the PET backbone or at the methyl ester formed by transesterification. Additionally, as the PET chain cleaves (by either ammonolysis or methanolysis), catalytic ethylene glycol is generated. The ethylene glycol can participate in the reaction as a catalyst and as a reactant via transesterification. Transesterification with glycol produces an ethyl-ester bond which provides another molecularly distinct site for ammonolysis.

**Figure 9:**
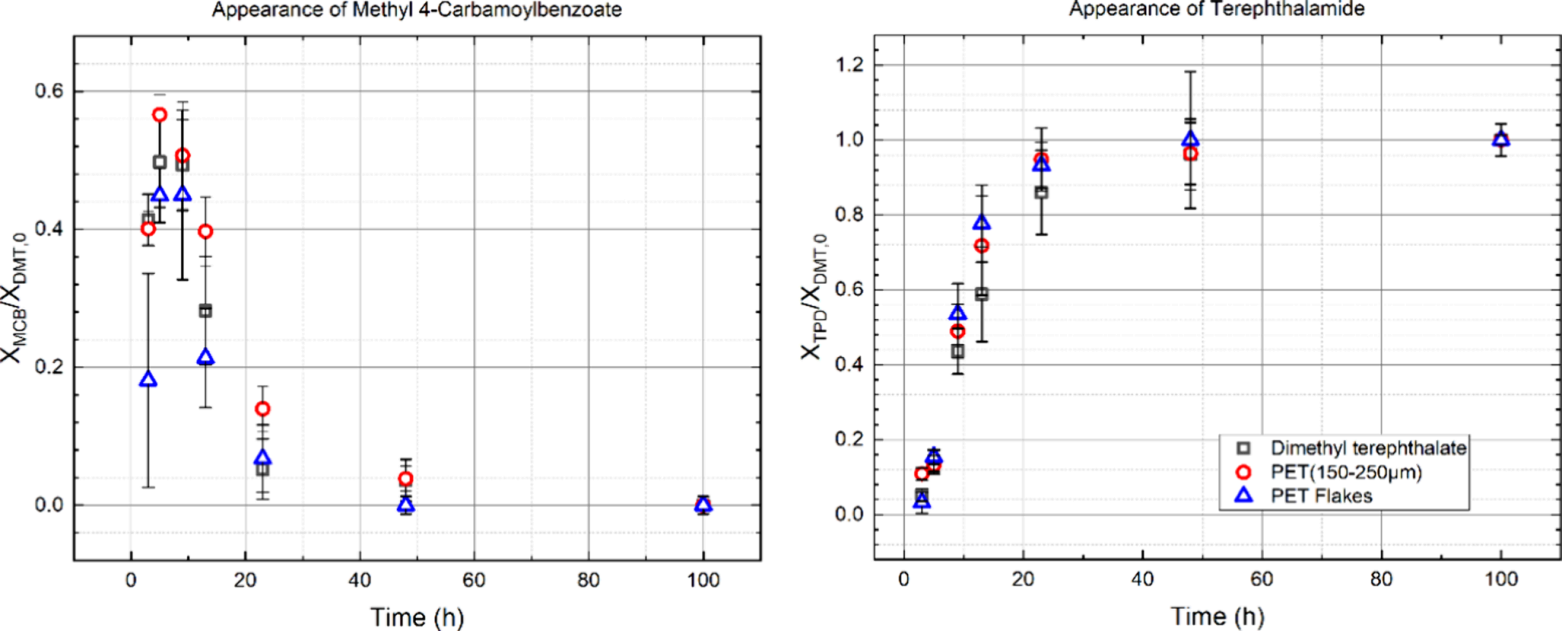
Production of MCB and TPD as a function of time at 100 °C using different feedstocks. Because ammonolysis of the ester linkage is rate limiting, differences are small.

Figure 9 shows the conversion of postconsumer PET waste to MCB and TPD. Despite the multiple possible pathways, small molecule production does not differ considerably from when DMT is used as the reactant. This finding is true when PET is used as either 1 cm^2^ flakes or as granulated 150–250 μm particles. One explanation of this experimental finding is that methanolysis proceeds much faster than ammonolysis. For example, supercritical methanol depolymerizes PET into DMT within 30 min at 200 °C.^29,31,33^ Even at the moderate temperature of 120 °C, PET is 98% converted to DMT in 2 h for a particle size of 128 μm.^32^ Comparison of the present findings to these previous studies establishes that the reaction rate of methanolysis is greater than the rate of ammonolysis. Accordingly, when PET ammonolysis is conducted in methanol, a significant fraction of the product is expected to pass through the methanolysis pathway. An alternative explanation is that on average all ester bonds, independent of type (PET backbone, methyl ester, ethyl ester), react at the same rate. Succinctly, ammonolysis of the ester linkage is the rate limiting step in all of the reaction pathways of Scheme 2.

In chemical recycling of plastics, mass transfer limitations are of great concern. When mass transfer limitations are present, conversion rates are controlled by diffusion of reactants into and products out of the piece of plastic material. The “rate limiting” step in the reaction pathway no longer limits the conversion rate when mass transfer rates are slow. Rate limiting mass transfer effects can generally be captured by studying conversion rates with particles of different sizes. PET flakes having a thickness of about 400 μm and granulated particles in the range of 150–250 μm are used as substrates to explore mass transfer limitations. Counterintuitively, despite the need for diffusion of methanol and ammonia into PET, its conversion rate to TPD was *faster* than that of DMT. This interesting finding is attributable to the autocatalytic effect of generating EG in situ during the ammonolysis of PET in methanol.

## 4 Conclusions

A comprehensive understanding of the ammonolysis of PET in methanol is now available based on controlled experimentation and mathematical modeling. For the model compound DMT, ammonia first attacks one of the two equivalent ester bonds to give the monoamide, methyl(4-carbamoylbenzoate) (MCB). Ammonia subsequently reacts with the second ester of MCB to yield the diamide (TPD). Each step consumes one ammonia and produces one methanol. A chemical kinetic model of the ammonolysis of the model DMT is presented which captures the observed experimental findings at a very high level of detail. Rate constants and activation energies are obtained by fitting the model to mole fractions as a function of time for reactions conducted at different temperatures. The catalytic effects of ethylene glycol on ammonolysis are unequivocally demonstrated. Reaction rates for the ammonolysis of DMT are compared to ammonolysis reaction rates when EG is present. It is demonstrated that a 3:1 molar ratio of EG:DMT increases the rate constants by more than 10-fold. A second detailed kinetic model is presented which does an excellent job of capturing these catalytic effects.

Application of the new understanding is employed to understand results of practicing ammonolysis on postuse thermoformed PET clamshell containers, which remain largely unrecovered and are usually landfilled. The autocatalytic effects of *in situ* generated ethylene glycol (EG) are clearly observed. The conversion rate of solid PET to terephthalamide (TPD) is demonstrated to proceed faster than the uncatalyzed conversion rate of DMT to TPD. That is, mass transfer limitations for the ammonolysis of thermoformed PET flakes are not rate limiting. The exposition of the catalytic effects of EG, along with other factors, indicate EG is the natural solvent medium for PET ammonolysis. The development of a detailed understanding of ammonolysis of PET in EG is the subject of part II of this work.

The present study demonstrates that ammonolysis may play an important role in the molecular recycling of PET. This is because it represents a facile modification to existing large-scale commercial practices. Specifically, commercial operations already conducting methanolysis and glycolysis can diversify their product streams easily, simply by dissolving ammonia into the methanol or glycol feed streams. The economic consequences of doing so is the subject of part III of this comprehensive work.

## Supporting Information Available

The Supporting Information includes the method for determining the mole fractions, thermochemistry calculations, solving of the differential equations, pictures of the reaction set up, temperature ramp figures, NMR figures, model fit figures, activity model figures, and Python code (PDF)

## ABBREVIATIONS

PET: poly(ethylene terephthalate)
DMT: dimethyl terephthalate
MCB: methyl 4-carbamoylbenzoate
TPD: terephthalamide
EG: ethylene glycol
