# Supplementary material for: Upcycling Waste PET: I. Ammonolysis Kinetics of Model Dimethyl Terephthalate and the Catalytic Effects of Ethylene Glycol
Source: ACS Sustain Chem Eng. 2025 Mar 6;13(10):4120–31. doi: 10.1021/acssuschemeng.4c10238 (PMC11921030; doi:10.1021/acssuschemeng.4c10238)
Supplement: Supplementary file 1 — sc4c10238_si_001.pdf [file sc4c10238_si_001.pdf]

Supporting Information for Publication

Upcycling waste PET: I. Ammonolysis kinetics of model dimethyl terephthalate and the catalytic effects of ethylene glycol

Richard-Joseph L. Peterson[1], Elanna P. Neppel[1], Lars Peereboom[1], P. Anh Trinh[1,2], Robert Y. Ofoli[1] and John R. Dorgan[1*]

37 pages, 19 figures, and 5 tables

1.0 Contents

1.0 Detailed Reaction Procedures 2
1.1 Reactor Cleaning and Assembly 2
1.2 Loading the reactants 3
1.3 Thermally quenching the reaction 3
2.0 Calculating mole fractions from NMR spectra 4
2.1 Example of determining mole fractions 7
3.0 Gibbs free energy of reaction 8
3.1 First reaction step 11
3.2 Second reaction step 12
3.3 Overall reaction 13
3.4 Calculation of Equilibrium Conversion 13
4.0 Solution of differential equations 14
4.1 DMT kinetics 15
4.2 DMT kinetics with ethylene glycol present 19
5.0 Supplemental figures 25
5.1 Model fit figures 25
5.3 NMR spectra 29
5.4 Activity model figure 35
6.0 Python Code to determine activation energy 36
7.0 References for supplemental materials 38

2.0 Detailed Reaction Procedures

1.1 Reactor Cleaning and Assembly

1. The reactor components are shown in Figure S1.
2. Prepare a solution of Alconox or other detergent solution according to the manufacturer's recommendations
3. Place the quartz insert into a quart size jar and cover with the cleaning solution (see Figure S2)
4. Place the reactor cylinder and head (with thermowell attached) into a metal canister of approximately quart size and cover with cleaning solution (see Figure S2)
5. Place the containers in the ultrasound bath and sonicate for 30 minutes (see Figure S2)
6. Rinse metallic components with acetone and the quartz with isopropyl alcohol, place in a dust and contaminant free environment to dry.

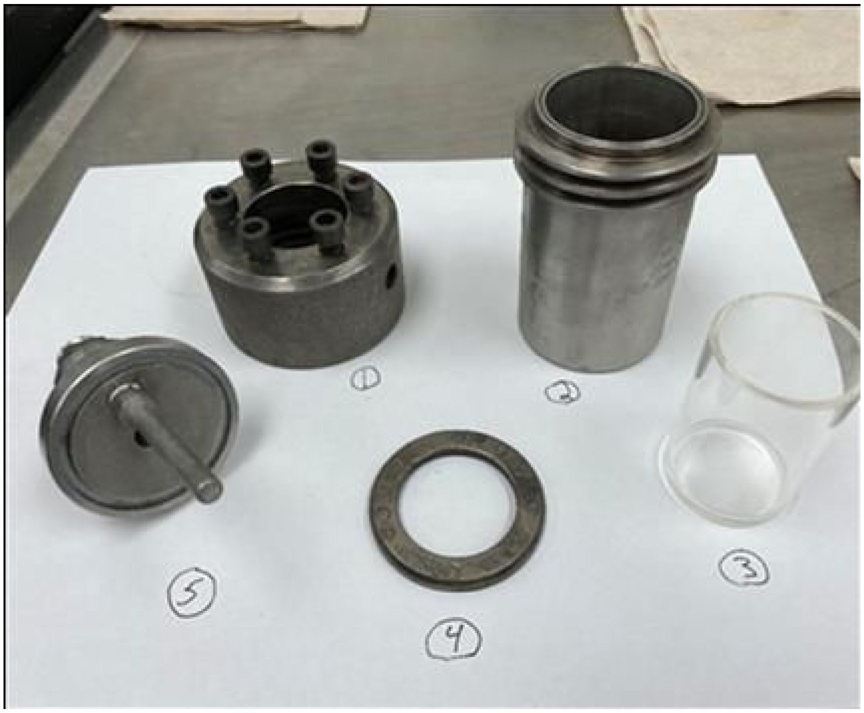

Figure S1. Reactor components (1. Collar with retention bolts, 2. Cylinder, 3. Quartz insert, 4. Retention ring, 5. Head with thermowell).

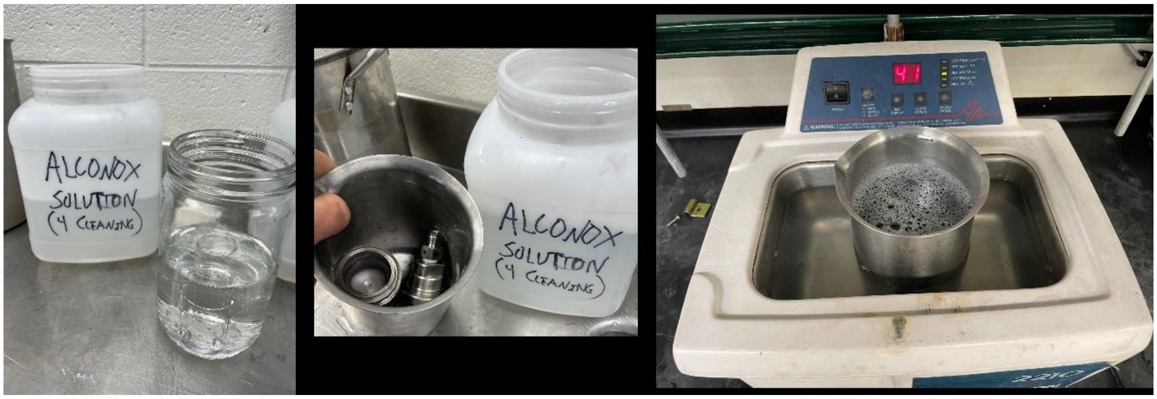

Figure S2. Cleaning of reaction contacting reactor components.

1.2 Loading the reactants

1. Charge 1-5 grams of solids or non-volatile liquids into the quartz liner and blanket with Argon gas.
2. Assemble the reactor, purge again with Argon gas and seal with rubber septum (see Figure S3). During assembly ensure the PTFE gasket is in place on the reactor head, that the head bolts are backed out, and that the compression ring is placed between the reactor head and collar bolts. Hand tighten the collar over the cylinder. Seal the reactor by tightening the hex bolts using a pattern that crosses over the center as each bolt is tightened to prevent misalignment.
3. Using a syringe, load the reactor vessel with 15-10 ml of liquid reactants (ammonia in methanol and ethylene glycol)
4. Place the reactor into the Parr 5000 multi-reactor system; make sure it is aligned in the heating mantle. Be sure to connect the correct thermocouple and gas inlet for the corresponding well location. Remove the rubber septum and connect the gas inlet; finger tighten the connection using a progressive forwards then backwards then forwards tightening to ensure no cross-threading. Simple finger tightening is sufficient.
5. Check that the nitrogen tank is open and that the regulator is adjusted to the desired pressure (typically 1200 psia) and that the vent valve on the manifold (the lower valve) is closed. Open the gas inlet valve (the upper valve on the Parr 5000 manifold) to allow the reactor to pressurize. Watch the pressure gauge for the corresponding reactor presented on the computer. When the reactor reaches the pressure of the nitrogen tank regulator, close the reactor inlet valve.
6. Wait for 1-2 minutes and note any changes in pressure. If the gauge pressure is unchanged or drops very little (a couple of psig) then proceed to the next step. Otherwise, slowly vent the remaining pressure, disassemble the reactor, and start the procedure over.
7. Turn on the corresponding temperature controller and enter the desired setpoint; manually adjust the agitation speed to the desired value (5-8 hundred RPM).
8. During the reaction, use the PC software will monitor and record the temperature, pressure and speed of rotation. Values may be observed in either the controller window or as a real time strip-chart type plot in the charting window.

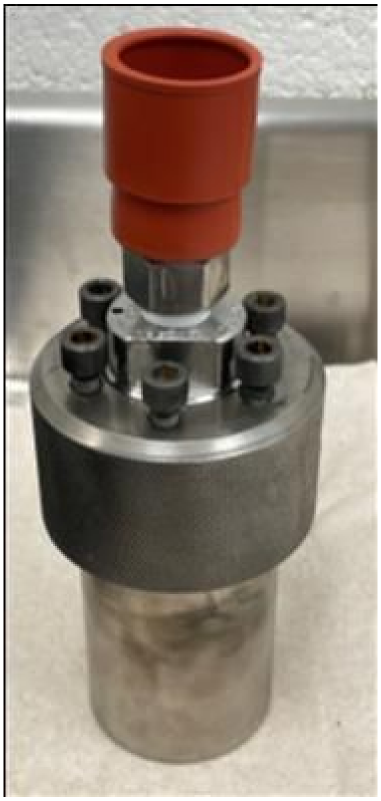

Figure S3. Assembled reactor, containing solids, purged with Argon gas, and fitted with a rubber septum to enable liquid charging using a syringe.

1.3 Thermally quenching the reaction

1. At the end of the desired reaction time, hit the large “STOP” button beneath the corresponding controller. Using heavy, heat resistant gloves, remove the reactor from the thermal well and place it in an ice bath contained by a metal pot (see Figure S4).

2. When the thermocouple reads 5⁰C, slowly release the pressure by opening the gas release valve (the lower valve on the manifold). Always wear heat resistant gloves, a flame and chemical resistant lab coat, and safety glasses - keep the hood sash as closed as possible.
3. Disconnect the reactor from the gas inlet and reattach rubber septum to avoid sample contamination. Dry the outside of the reactor with cloth or paper towels.
4. Carefully disassemble the reactor. Note: because the reactor is now cold and the metallic components have shrunk, disassembly should be easy. If fittings are tight, double check your procedures. Bolts may be loosened using the vice and a wrench, but final disassembly should be by hand and in a chemical hood.
5. Remove the glass insert and drain any liquids remaining in the metal cylinder into a sample collection container. Retain both linear and spillover for subsequent product work up. Remove the other reactor components from the hood and place out of the way for subsequent cleaning.

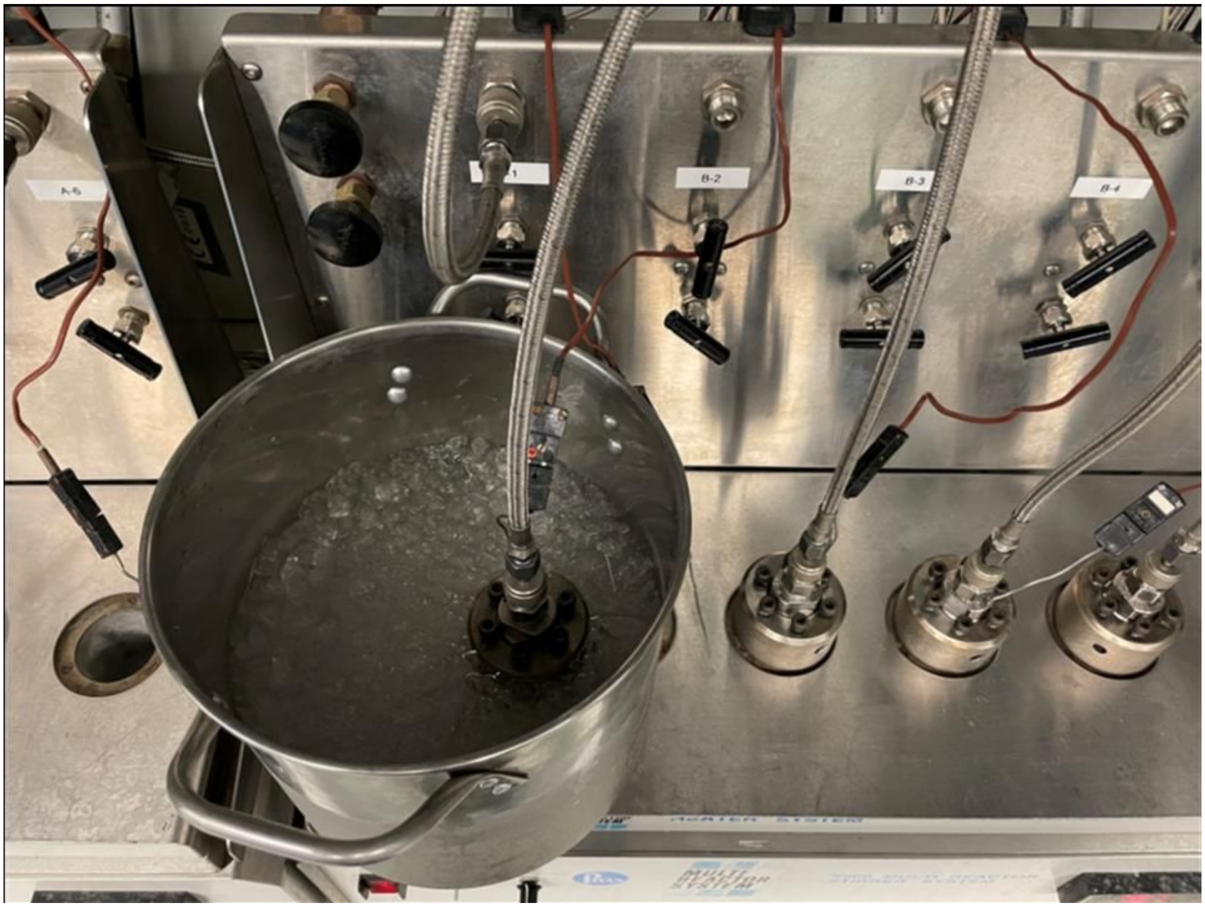

Figure S4. Thermal quenching in an ice bath.

3.0 2.0 Calculating mole fractions from NMR spectra

The integrals of the NMR peaks in the aromatic region (approximately 6.5PPM to 8.5PPM) are used to determine how much of each species is present in the recovered solids. The aromatic hydrogens are the best metric to use to compare the relative amounts of each of the compounds because each compound has the same number (4). The aromatic peak locations are discussed in the Results section of the paper. If there is overlap between two peaks, Eqn. S1 is used to calculate the integral of the desired peak.

$$I_{DMT} = \int_a^b f(x)dx - \int_c^d f(x)dx \quad \text{(S1)}$$

Here, f(x) represents the NMR curve. The interval a to b defines the boundaries of the overlapping peaks. Whereas the interval c to d specifies the bounds of a separate peak, which has a signal with an equivalent number of protons responsible for the undesired portion of the overlapping peaks. For example, in Figure S5 the peak at 8.06 PPM is an overlap of the aromatic hydrogens of dimethyl terephthalate (DMT) and two amide hydrogens of terephthalamide (TPD). In this example, a is 8.05PPM and b is 8.10PPM. There is another peak at 7.49PPM that is the other two amide hydrogens of TPD. Additionally, c is 7.47PPM and d is 7.52PPM. The resulting value, I_{DMT}, is the true integral of the dimethyl terephthalate peak.

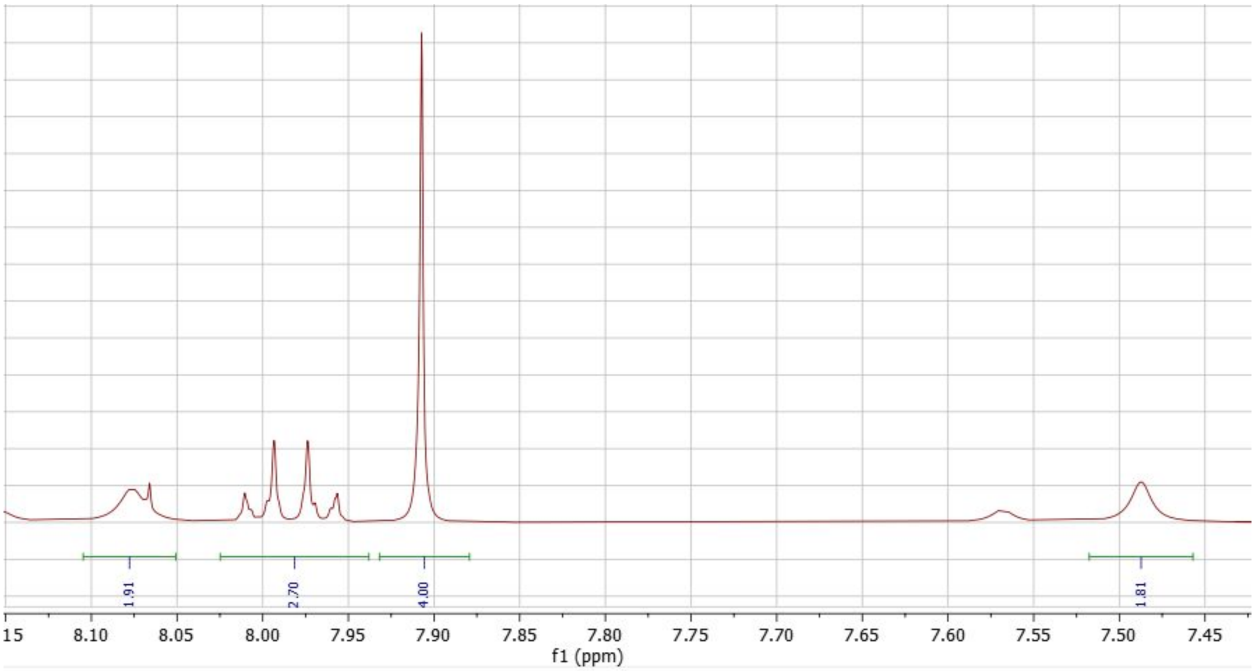

Figure S5. Representative 1H NMR between 7.45 and 8.15PPM for 13 h reaction time at 100°C. The spectrum is zoomed into the aromatic region to illustrate the analysis. Terephthalamide 1H NMR ((500MHz, DMSO) δ 8.06 (s, 2H), 7.91 (s, 4H), 7.49 (s, 2H)), Dimethyl Terephthalate 1H NMR ((500 MHz, DMSO) δ 8.06 (s, 4H), 3.87 (s, 6H)), and Methyl 4 carbamoylbenzoate (6757-31-9) 1H NMR ((500 MHz, DMSO) δ 8.15 (s, 1H), 8.03 – 7.93 (m, 4H), 7.56 (s, 1H), 3.85 (s, 3H)).

The molar ratios $R_{dry,\,DMT}$, $R_{dry,MCB}$, and $R_{dry,TPD}$ of each of DMT, methyl 4-carbamoylbenzoate (MCB), and TPD, respectively were calculated using Eqns. S2-S4.

$$R_{dry,\,DMT} = \frac{I_{DMT}}{I_{DMT} + I_{MCB} + I_{TPD}} \tag{S2}$$

$$R_{dry,MCB} = \frac{I_{MCB}}{I_{DMT} + I_{MCB} + I_{TPD}} \tag{S3}$$

$$R_{dry,TPD} = \frac{I_{TPD}}{I_{DMT} + I_{MCB} + I_{TPD}} \tag{S4}$$

Here I_{MCB} and I_{TPD} are the integrals in the aromatic region of the NMR spectrum of MCB and terephthalamide TPD, respectively.

The initial moles of the solid reactant (DMT) were determined by Eqn. S5.

$$n_{initial,\,DMT} = \frac{m_{initial,DMT}}{M_{w,DMT}} \tag{S5}$$

Where $m_{initial,\,DMT}$is the mass of DMT loaded into the reactor and $M_{w,DMT}$ is the molecular weight of DMT.

The ammonia solution, present in the reaction, needed to be accounted for to determine the mole fractions. The known molarity of the solution was converted into a mole fraction of the solute in solution using Eqn. S6.

$$M_{w,solute} * C * V = m_{solute} \tag{S6}$$

In Eqn. S6, M_w is the molecular weight of the compound in g/mol, *C* is the concentration, *V* the volume of the solution used, and *m* is the mass of the compound in that solution. The mass of the solvent is given by,

$$m_{solvent} = \rho * V - m_{solute} \tag{S7}$$

where ρ is the density of the solution. The mass of the solvent and solute were converted to moles using,

$$n_{solvent} = \frac{m_{solvent}}{M_{w,solvent}} \tag{S8}$$

$$n_{solute} = \frac{m_{solute}}{M_{w,solute}} \tag{S9}$$

where $M_{w,solvent}$ is the molecular weight of the solvent and $M_{w,solute}$ is the molecular weight of the solute. In this study, the solvent is methanol and the solute is ammonia as shown in Eqns. S10-S11.

$$n_{solvent} = n_{methanol} \tag{S10}$$

$$n_{solute} = n_{ammonia} \tag{S11}$$

The mole fractions of ammonia ($x_{ammonia}$) and the solids (x_{solids}) were determined by dividing the initial moles of ammonia by the sum of the moles in the reactor using Eqns. S12-S13.

$$x_{ammonia} = \frac{n_{ammonia}}{n_{ammonia} + n_{methanol} + n_{initial,DMT}} \tag{S12}$$

$$x_{solids} = \frac{n_{initial,DMT}}{n_{ammonia} + n_{methanol} + n_{initial,DMT}} \tag{S13}$$

It is assumed that the mole fraction of ammonia are constant because ammonia is present in large excess (approximately 25 times the stoichiometric amount). The molar ratios of the solids, as determined using NMR spectroscopy, are multiplied by the solid mole fractions to obtain individual mole fractions at each time step using Eqns. S14-S16.

$$x_{DMT} = x_{solids} * R_{dry,\,DMT} \tag{S14}$$

$$x_{MCB} = x_{solids} * R_{dry,\,MCB} \tag{S15}$$

$$x_{TPD} = x_{solids} * R_{dry,\,TPD} \tag{S16}$$

The mole fractions as a function of time at each average temperature constitute the primary data.

2.1 Example of determining mole fractions

An example of determining the mole fractions using Eqns. S1-S16 and the values in Figure S5 follows.

$$I_{DMT} = \int_{8.05ppm}^{8.10ppm} f(x)dx - \int_{7.47ppm}^{7.52ppm} f(x)dx = 1.91 - 1.81 = 0.10 \tag{S17}$$

The resulting value, I_{DMT}, is the integral of the dimethyl terephthalate peak. The integrals of MCB and TPD are 2.70 and 4.00, respectively. The integrals were added together to get the total moles of aromatic hydrogens. The integrals for each aromatic peak were divided by the total moles of aromatic hydrogens to get a dry mole fraction.

$$R_{dry,\,DMT} = \frac{0.10}{0.10 + 2.70 + 4.00} = .015 \tag{S18}$$

$$R_{dry,MCB} = \frac{2.70}{0.10 + 2.70 + 4.00} = .397 \tag{S19}$$

$$R_{dry,TPD} = \frac{4.00}{0.10 + 2.70 + 4.00} = .588 \tag{S20}$$

The known molarity of the solution was multiplied by the molar mass of ammonia to get the number of grams of ammonia in a mL of solution.

$$17.03g/mol * 7\ mol/L = .119\ g/mL \tag{S21}$$

A 1.0 mL basis was used to determine the number of moles of methanol in a mL of the solution. In 1 mL there is .779 g of solution and .119 g of ammonia.

$$m_{solvent} = m_{methanol} = .779g - .119g = .660\ g \quad \text{(S22)}$$

$$n_{methanol} = \frac{.660g}{32.04\ g/mol} = 20.6\ mmol \quad \text{(S23)}$$

$$n_{ammonia} = \frac{.119g}{17.04\ g/mol} = 7\ mmol \quad \text{(S24)}$$

The mmols of ammonia and methanol present in the reactor were determined by multiplying the molarity by 15 mL.

$$n_{methanol} = 20.6\ mmol/mL * 15mL = 309\ mmol \quad \text{(S25)}$$

$$n_{ammonia} = 7\ mol/L * 15\ mL = 105\ mmol \quad \text{(S26)}$$

The initial number of moles of DMT were calculated by dividing the initial mass of DMT by the molecular weight of DMT.

$$n_{initial,\,DMT} = \frac{1.00g}{194.19g/mol} = 5.15mmol \quad \text{(S27)}$$

The mole fraction of ammonia was determined by dividing the initial moles of ammonia by the sum of the moles in the reactor.

$$x_{ammonia} = \frac{105mmol}{105mmol + 309mmol + 5.15mmol} = .251 \quad \text{(S28)}$$

$$x_{solids} = \frac{5.15mmol}{105mmol + 309mmol + 5.15mmol} = .0123 \quad \text{(S29)}$$

The molar ratios of the solids, as determined using NMR spectroscopy, were multiplied by the solid mole fractions to obtain individual mole fractions.

$$x_{DMT,13h,100^oC} = .0123 * .015 = .00019 \quad \text{(S30)}$$

$$x_{MCB,13h,100^oC} = .0123 * .397 = .00488 \quad \text{(S31)}$$

$$x_{TPD,13h,100^oC} = .0123 * .588 = .00723 \quad \text{(S32)}$$

3.0 Gibbs free energy of reaction

Thermodynamic equilibrium for the reaction was evaluated by determining the Gibbs free energy of reaction. This estimation requires the Gibbs free energy of formation of each compound. The NIST Webbook (https://webbook.nist.gov/chemistry/) provides Gibbs free energy of formation values for ammonia and methanol. The reported Gibbs free energy of formation for Ammonia (gas) and methanol (liquid) are -16.4 kJ/mol and -166.6 kJ/mol respectively.

Since the value of the Gibbs energy of formation is given for ammonia as a gas and ammonia will be dissolved in methanol as a liquid, the value must be converted; this is done using Eqn. S33.

$$\Delta G_f^{liq}(298, 1bar) = \Delta G_f^{gas} + V_L\left(1 - P_{298}^{SAT}\right) + RTln\left(\frac{P_{298}^{SAT}}{1bar}\right) \quad \text{(S33)}$$

ΔG_f^{liq} is the Gibbs free energy of formation in the liquid phase, ΔG_f^{gas} is the Gibbs free energy of formation in the gas phase, V_L is molar volume, P_{298}^{SAT} is the saturation pressure at 298K, R is the ideal gas constant, and T is absolute temperature. The saturation pressure at 298K is shown in Eqn. S34.

$$P_{298}^{SAT} = 10bar \quad \text{(S34)}$$

The density of liquid ammonia is expressed in Eqn. S35.

$$\rho = 0.73\,\frac{g}{cm^3}\left(10^6\frac{cm^3}{m^3}\right)\left(\frac{1kg}{10^3g}\right) = 730\ kg/m^3 \quad \text{(S35)}$$

The molar volume of ammonia is calculated by dividing the molecular weigh by the density of ammonia in Eqn. S36.

$$V_L = \frac{1}{730\,\frac{kg}{m^3}}\,17\,\frac{g}{mol}\left(\frac{1kg}{10^3g}\right) = 2.33 * 10^{-5}m^3/mol \quad \text{(S36)}$$

The volume portion of Eqn. S33 was calculated in Eqn. S37.

$$V_L\left(1 - P_{298}^{SAT}\right) = 2.33 * 10^{-5}\frac{m^3}{mol}\left(1 * 10^5\frac{N}{m^2} - 10 * 10^5\frac{N}{m^2}\right) = -2.1\,\frac{Nm}{mol} = 2 * 10^{-3}kJ/mol \quad \text{(S37)}$$

The pressure portion of Eqn. S33 is then used in Eqn. S38.

$$RTln\left(\frac{P_{298}^{SAT}}{1bar}\right) = 2.48\,\frac{kJ}{mol} * ln\left(\frac{10bar}{1bar}\right) = 5.71\ kJ/mol \quad \text{(S38)}$$

Eqns. S37-S38 are substituted into Eqn. S33 resulting in Eqn. S39.

$$\Delta G_{f,ammonia}^{liq}(298, 1bar) = -16.4\,\frac{kJ}{mol} + .002\,\frac{kJ}{mol} + 5.71\,\frac{kJ}{mol} = \mathbf{-10.69\ kJ/mol} \quad \text{(S39)}$$

To determine Gibbs free energy of formations for species not in the NIST Webbook, the group contribution developed by L. Constantinou is employed [1]. This method uses Eqn. S40.

$$\Delta G_f = g_0 + \sum N_j g_{1j} + \sum M_j g_{2j} \quad \text{(S40)}$$

Where g_0 is a constant, g_{1j} is the Gibbs free energy of formation of primary functional group *j*, N_j is the number of occurrences of primary functional group *j*, g_{2j} is the Gibbs free energy of formation of primary functional group *j*, and M_j is the number of occurrences of primary functional group *j*.

Table S1. The group contribution calculation of Gibbs energy of formation for DMT

Dimethyl terephthalate (DMT)

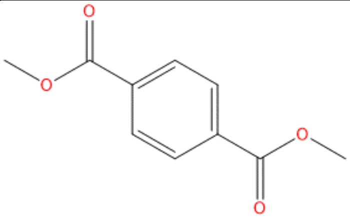

Primary Group	# of occurrences	g_{ij} (kJ/mol)	Contribution (kJ/mol)
CH_3	2	-8.030	-16.060
COO	2	-281.495	-562.990
AC	2	30.485	60.970
ACH	4	22.533	90.132
Secondary group			
ACOO	2	-7.415	-14.830
Constant			
g_0	1	-14.828	-14.828
ΔG_f^{DMT}	-	-	**-457.61**

Table S2. The group contribution calculation of Gibbs energy of formation for MCB.

Methyl 4-carbamoylbenzoate (MCB)

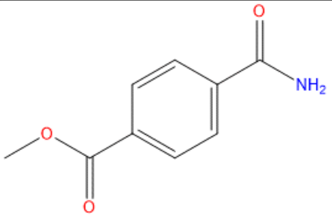

Primary Group	# of occurrences	g_{ij} (kJ/mol)	Contribution (kJ/mol)
CH_3	1	-8.030	-8.030
COO	1	-281.495	-281.495
$CONH_2$	1	-136.742	-136.742
AC	2	30.485	60.970
ACH	4	22.533	90.132
Secondary group			
ACOO	1	-7.415	-7.415
Constant			
g_0	1	-14.828	-14.828
ΔG_f^{DMT}	-	-	**-297.41**

Table S3. The group contribution calculation of Gibbs energy of formation for TPD.

Terephthalamide (TPD)	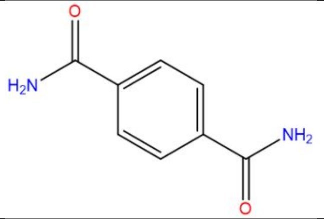		
Primary Group	# of occurrences	g_{ij} (kJ/mol)	Contribution (kJ/mol)
$CONH_2$	2	-136.742	-136.742
AC	2	30.485	60.970
ACH	2	22.533	90.132
Constant			
g_0	1	-14.828	-14.828
ΔG_f^{DMT}	-	-	**-137.21**

3.1 First reaction step

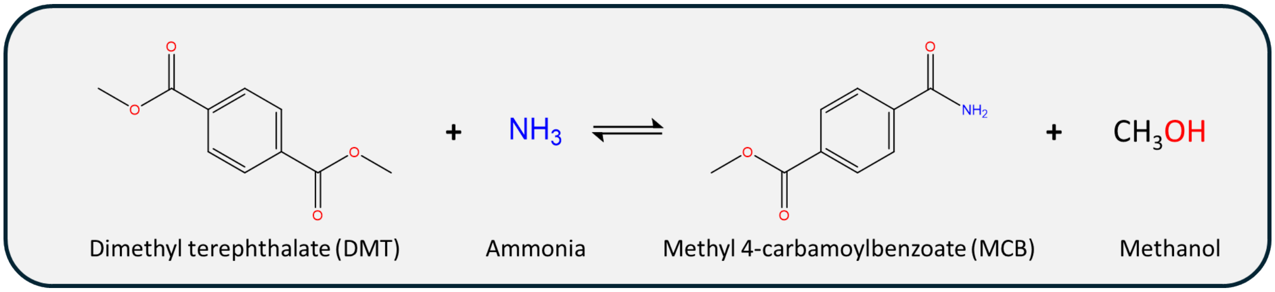

Scheme S1. The first reaction step expressed as an equilibrium reaction

The Gibbs free energy of reaction can be determined using Eqn. S41.

$$\Delta G_{rxn} = \sum \nu_i \Delta G_f^i = \left(-297.41\frac{kJ}{mol} - 181.43\frac{kJ}{mol}\right) - \left(-457.61\frac{kJ}{mol} - 10.69\frac{kJ}{mol}\right) \quad \text{(S41)}$$

Where ν_i is the stoichiometric coefficient for species *i* and ΔG_f^i is the Gibbs energy of formation for species *i*. The resulting Gibbs free energy of reaction for the first reaction step is given in Eqn. S42.

$$\Delta G_{rxn1} = -10.54\ kJ/mol \quad \text{(S42)}$$

The Gibbs energy of reaction can be related to the equilibrium constant by Eqns. S43-S44.

$$\Delta G_{rxn} = -RT lnK \quad \text{(S43)}$$

$$K = \exp\left(-\frac{\Delta G_{rxn}}{RT}\right) \quad \text{(S44)}$$

At T=25^oC the equilibrium constant is calculated using Eqn. S45.

$$K = \exp\left(\frac{10.54\,\frac{kJ}{mol}}{0.008314\,\frac{kJ}{molK} * 298.15K}\right) \tag{S45}$$

$$K = 70.1 \tag{S46}$$

At T=100°C the equilibrium constant is,

$$K = \exp\left(\frac{10.54\,\frac{kJ}{mol}}{0.008314\,\frac{kJ}{molK} * 373.15K}\right) \tag{S47}$$

$$K = 30.0 \tag{S48}$$

3.2 Second reaction step

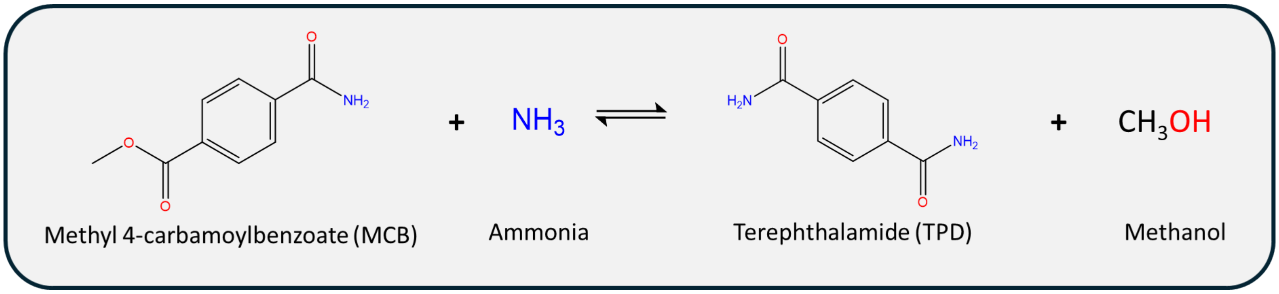

Scheme S2. The second reaction step expressed as an equilibrium reaction

The Gibbs free energy of the second reaction step can be determined with Eqn. S49. The result is the same value because both reactions are the same in terms of functional groups.

$$\Delta G_{rxn} = \sum \nu_i \Delta G_f^i = \left(-137.21\,\frac{kJ}{mol} - 181.43\,\frac{kJ}{mol}\right) - \left(-297.41\,\frac{kJ}{mol} - 10.69\,\frac{kJ}{mol}\right) \tag{S49}$$

$$\Delta G_{rxn2} = -10.54\ kJ/mol \tag{S50}$$

The Gibbs free energy of reaction can be related to the equilibrium constant by Eqns. S51-S52.

$$\Delta G_{rxn} = -RT\ln K \tag{S51}$$

$$K = \exp\left(-\frac{\Delta G_{rxn}}{RT}\right) \tag{S52}$$

At T=25°C the equilibrium constant was found in Eqns. S53-S54.

$$K = \exp\left(\frac{10.54\,\frac{kJ}{mol}}{0.008314\,\frac{kJ}{molK} * 298.15K}\right) \tag{S53}$$

$$K = 70.1 \tag{S54}$$

At T=100°C the equilibrium constant was found in Eqns. S55-S56.

$$K = \exp\left(\frac{10.54\,\frac{kJ}{mol}}{0.008314\,\frac{kJ}{molK} * 373.15K}\right) \tag{S55}$$

$$K = 30.0 \tag{S56}$$

3.3 Overall reaction

To determine the Gibbs free energy of the overall reaction, the two Gibbs free energies are summed. The product is taken of the two equilibrium constants to determine the overall equilibrium constant resulting in Eqns. S57-S59.

$$\Delta G_{rxn} = -21.08\ kJ/mol \tag{S57}$$

$$K_{overall,\,25^{o}C} = 70.1^2 = 4910 \tag{S58}$$

$$K_{overall,100^{o}C} = 30.0^2 = 900 \tag{S59}$$

3.4 Calculation of Equilibrium Conversion

Let y be the moles of DMT reacted (i.e. conversion)

Table S4. The number of moles of each compound at time=0 and at equilibrium

Compound	At time=0 (mol)	At Equilibrium (mol)
Dimethyl terephthalate (DMT)	1	$N_{DMT} = 1 - y$
Terephthalamide (TPD)	0	$N_{TPD} = y$
Ammonia	19	$N_{Ammonia} = 19 - 2y$
Methanol	57	$N_{methanol} = 57 + 2y$
Total	77	77

The equilibrium is expressed as a function of the activities of the products and reactants in Eqn. S60.

$$K_{overall} = \frac{a_{TPD}a_{methanol}^2}{a_{DMT}a_{ammonia}^2} \tag{S60}$$

Assuming an Ideal solution (the activity is assumed to be equal to the mole fraction) as seen in Eqn. S61.

$$K_{overall} = \frac{x_{TPD}x_{methanol}^2}{x_{DMT}x_{ammonia}^2} \tag{S61}$$

Substituting the mole fractions from Table S4 into Eqn. S61 results in Eqn. S62.

$$K_{overall} = \frac{\frac{y}{77}\left(\frac{57+2y}{77}\right)^2}{\frac{1-y}{77}\left(\frac{19-2y}{77}\right)^2} \quad \text{(S62)}$$

Simplifying and rearranging result in Eqns. S63-S65.

$$K_{overall} = \frac{y(57+2y)^2}{(1-y)(19-2y)^2} \quad \text{(S63)}$$

$$K_{overall}(1-y)(19-2y)^2 = y(57+2y)^2 \quad \text{(S64)}$$

$$K_{overall}(1-y)(19-2y)^2 - y(57+2y)^2 = 0 \quad \text{(S65)}$$

The $K_{overall,100^oC} = 900$ from Eqn. S59 value is placed in for $K_{overall}$ resulting in Eqn. S66.

$$900(1-y)(19-2y)^2 - y(57+2y)^2 = 0 \quad \text{(S66)}$$

Now that the equation is in residual form, the equation can be solved numerically (Mathematica was used).

$$y^{equil} = 0.987 \quad \text{(S67)}$$

The results of the estimations for the conversion are consistent with the high yields obtained experimentally.

4.0 4.0 Solution of the coupled ordinary differential equations

The differential equations comprising the chemical kinetics model were solved using Laplace transforms.

Table S5 shows a list of chemicals and their name and letter and reaction scheme.

Table S5. Chemicals names and structures with the corresponding variable used for their mole fraction in the differential equation.

**Chemical Structure**	**Name**	**Variable name of species**
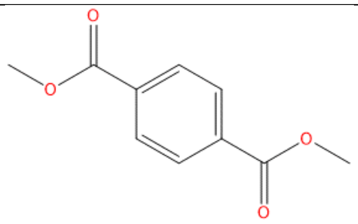	Dimethyl terephthalate	A

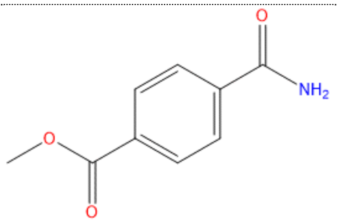	Methyl 4-carbamoylbenzoate	B
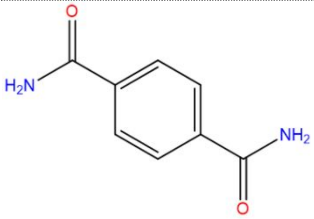	Terephthalamide	C
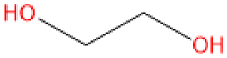	Ethylene Glycol	E
NH_3	Ammonia	F
CH_3OH	Methanol	G

4.1 DMT kinetics

The differential equations for the DMT kinetics comprise Eqns. S68-S70

$$\frac{dA}{dt} = -k_1AF \tag{S68}$$

$$\frac{dB}{dt} = k_1AF - k_2BF \tag{S69}$$

$$\frac{dC}{dt} = k_2BF \tag{S70}$$

Where k_1 and k_2 are the rate constants for the first and second reaction steps respectively and A, B, C, and F are the mole fraction of DMT, MCB, TPD, and ammonia respectively. The concentration of ammonia (F) and methanol (G) are assumed to be constant in Eqns. S71-S72.

$$\frac{dF}{dt} = 0 \tag{S71}$$

$$\frac{dG}{dt} = 0 \tag{S72}$$

The equations are solved in Laplace space, the transform of Eqn. S68 is

$$sA(s) - A_0 = -k_1A(s)F \tag{S73}$$

$$A(s) = \frac{A_0}{s + k_1F} \tag{S74}$$

where s is the Laplace space variable and A_0 is the initial mole fraction of DMT. The final expression is shown in Eqn. S75.

$$A(t) = A_0\exp\left(-k_1Ft\right) \tag{S75}$$

Eqn. S69 was transformed into the Laplace domain to give S76

$$sB(s) - B_0 = k_1A(s)F - k_2B(s)F \tag{S76}$$

The appropriate initial condition is given by S77,

$$B_0 = 0 \tag{S77}$$

yielding Eqn. S78.

$$sB(s) = k_1\frac{A_0}{s + k_1F}F - k_2B(s)F \tag{S78}$$

Rearrangement gives,

$$B(s) = \frac{\left(\frac{k_1A_0F}{s + k_1F}\right)}{s + k_2F} \tag{S79}$$

$$B(s) = \frac{k_1A_0F}{(s + k_1F)(s + k_2F)} \tag{S80}$$

Partial fraction expansion of S80 gives S81

$$B(s) = \frac{X}{s} + \frac{Y}{s + k_1F} + \frac{Z}{s + (k_2F)} \tag{S81}$$

Where X, Y, and Z are the numerators to be solved for in the partial fraction expansion. For simplification, a_1 and a_2 are defined in Eqns. S82-S83.

$$a_1 = k_1F \tag{S82}$$

$$a_2 = k_2F \tag{S83}$$

Substituting a_1 and a_2 and setting X to 0 gives Eqn. S84.

$$\frac{k_1A_0F}{(s + k_1F)(s + k_2F)} = \frac{Y}{s + a_1} + \frac{Z}{s + a_2} \tag{S84}$$

Determining the common denominator, and multiplying it to both sides of Eqn. S84 gives Eqn. S85.

$$k_1A_0F = Y(s + a_2) + Z(s + a_1) \tag{S85}$$

Distributing Y and Z and factoring s, results in Eqn. S86.

$$s * 0 + k_1 A_0 F = s(Y + Z) + (Ya_2 + Za_1) \quad \text{(S86)}$$

Separating into an s term and a constant term yields Eqns. S87-S88

s term	$Y + Z = 0$	(S87)
Constant term	$Ya_2 + Za_1 = k_1 A_0 F$	(S88)

Solving for Z in S87 gives S89.

$$Z = -Y \quad \text{(S89)}$$

Plugging Eqn. S89 into Eqn. S88, results in S90.

$$k_1 A_0 F = Ya_2 + (-Y)a_1 \quad \text{(S90)}$$

Algebraically solving for Y gives Eqn. S91.

$$\frac{(k_1 A_0 F)}{a_2 - a_1} = Y \quad \text{(S91)}$$

Plugging S82-S83 into S91 results in S92.

$$Y = \frac{k_1 A_0 F}{k_2 F - k_1 F} \quad \text{(S92)}$$

Substituting Eqn. S92 into Eqn S89 gives Eqn. S93.

$$Z = -\frac{k_1 A_0 F}{k_2 F - k_1 F} \quad \text{(S93)}$$

The inverse Laplace transform of S81 is S94.

$$B(t) = X + Yexp(-k_1 Ft) + Zexp(-k_2 Ft) \quad \text{(S94)}$$

Plugging the values obtained for X, Y, and Z into S94 and simplifying yields the solution in Eqn. S95

$$B(t) = \frac{k_1 A_0}{k_2 - k_1} exp(-k_1 Ft) - \frac{k_1 A_0}{k_2 - k_1} exp(-k_2 Ft) \quad \text{(S95)}$$

Eqn. S70 was transformed into the Laplace domain and C(s) was solved for in Eqns. S96,

$$sC(s) - C_0 = k_2 B(s) F \quad \text{(S96)}$$

Where C_0 is the initial mole fraction of TPD. An appropriate initial mole fraction of 0 was chosen,

$$C_0 = 0 \quad \text{(S97)}$$

yielding Eqn. S98.

$$C(s) = \frac{k_2 B(s) F}{s} \quad \text{(S98)}$$

Plugging Eqn. S80 into Eqn. 98 and subsequent rearrangement results in Eqns. S99-S100.

$$C(s) = \frac{k_2\left(\frac{k_1 A_0 F}{(s + k_1 F)(s + k_2 F)}\right)F}{s} \tag{S99}$$

$$C(s) = \frac{k_1 k_2 A_0 F^2}{s(s + k_1 F)(s + k_2 F)} \tag{S100}$$

Partial fraction expansion of S100 gives S101.

$$\frac{k_1 k_2 A_0 F^2}{s(s + k_1 F)(s + k_2 F)} = C(s) = \frac{X}{s} + \frac{Y}{s + k_1 F} + \frac{Z}{s + k_2 F} \tag{S101}$$

For simplification, a_1 and a_2 are defined in Eqns. S102-S103.

$$a_1 = k_1 F \tag{S102}$$

$$a_2 = k_2 F \tag{S103}$$

Determining the common denominator, and multiplying it to both sides of Eqn. S101 gives Eqn. S104.

$$k_1 k_2 A_0 F^2 = X(s + a_1)(s + a_2) + Y(s + a_2)s + Z(s + a_1)s \tag{S104}$$

Distributing results in S105.

$$k_1 k_2 A_0 F^2 = Xs^2 + Xsa_1 + Xsa_2 + Xa_1 a_2 + Ys^2 + Ysa_2 + Zs^2 + Zsa_1 \tag{S105}$$

Separating S105 into s^2, s, and constant terms yields Eqns. S106-S108.

s^2 term
$$X + Y + Z = 0 \tag{S106}$$

s term
$$X(a_1 + a_2) + Ya_2 + Za_1 = 0 \tag{S107}$$

Constant term
$$k_1 k_2 A_0 F^2 = Xa_1 a_2 \tag{S108}$$

Solving for X in Eqn. S108 and substituting Eqns. S102-S103 results in Eqn. S109.

$$X = \frac{k_1 k_2 A_0 F^2}{a_1 a_2} = \frac{k_1 k_2 A_0 F^2}{k_1 F(k_2 F)} = A_0 \tag{S109}$$

Substituting S109 into S106 yields S110.

$$A_0 + Y + Z = 0 \tag{S110}$$

Solving for Z results in Eqn. S111.

$$Z = -A_0 - Y \tag{S111}$$

Eqns. S111 and S109 are substituted into Eqn. S107 to yield Eqn. S112.

$$0 = A_0(a_1 + a_2) + Ya_2 + (-A_0 - Y)a_1 \tag{S112}$$

Rearrangement results in Eqns. S113-S114.

$$-A_0(a_1+a_2)+A_0a_1=Ya_2-Ya_1 \tag{S113}$$

$$\frac{(-A_0a_2)}{a_2-a_1}=Y \tag{S114}$$

Substituting Eqns. S102-S103 into S114 gives Eqn. S115.

$$Y=\frac{-k_2A_0}{k_2-k_1} \tag{S115}$$

Substituting Eqn. S115 into Eqn. S111 yields Eqn. S116.

$$Z=\frac{k_2A_0}{k_2-k_1}-A_0 \tag{S116}$$

Rearrangement of Eqn. S116 shown in Eqns. S117-S118 results in Eqn. S119.

$$Z=A_0\left(\frac{k_2}{k_2-k_1}-1\right) \tag{S117}$$

$$Z=A_0\left(\frac{k_2}{k_2-k_1}-\frac{k_2-k_1}{k_2-k_1}\right) \tag{S118}$$

$$Z=A_0\left(\frac{k_1}{k_2-k_1}\right) \tag{S119}$$

The inverse Laplace transform of Eqn. S101 results in Eqn. S121.

$$C(t)=X+Yexp(-k_1Ft)+Zexp(-k_2Ft) \tag{S121}$$

Substituting Eqns. S109, S115, and S119 into Eqn. S121 yields Eqn. S122.

$$C(t)=A_0+\frac{-k_2A_0}{k_2-k_1}exp(-k_1Ft)+A_0\left(\frac{k_1}{k_2-k_1}\right)exp(-k_2Ft) \tag{S122}$$

4.2 DMT kinetics with ethylene glycol present

Let k_3 and k_4 be the rate constants for the catalyzed pathway for the first and second steps of the reaction respectively, while k_1 and k_2 are the uncatalyzed rates constants for those steps. The resulting chemical kinetic model is given by S123-S125.

$$\frac{\mathrm{dA}}{\mathrm{dt}}=-k_1AF-k_3AEF \tag{S123}$$

$$\frac{dB}{dt}=k_1AF+k_3AEF-k_2BF-k_4BEF \tag{S124}$$

$$\frac{dC}{\mathrm{dt}}=k_2BF+k_4BEF \tag{S125}$$

Where k_1 and k_2 are the rate constants for the uncatalyzed first and second reaction steps respectively, k_3 and k_4 are the rate constants for the catalyzed first and second reaction steps respectively, and A, B, C, E, and F are the mole fraction of DMT, MCB, TPD, ethylene glycol, and ammonia respectively. The concentration of ammonia is assumed to be constant.

The concentration of ammonia, methanol and ethylene glycol are assumed to be constant, resulting in Eqns. S126-S128

$$\frac{dF}{dt} = 0 \tag{S126}$$

$$\frac{dG}{dt} = 0 \tag{S127}$$

$$\frac{dE}{dt} = 0 \tag{S128}$$

Where G is the mole fraction of methanol. Eqn. S123 was solved in Laplace space resulting in Eqns. S129-S130

$$sA(s) - A_0 = -\,k_1 A(s)F - k_3 A(s)EF \tag{S129}$$

$$A(s) = \frac{A_0}{s + (k_1 F + k_3 EF)} \tag{S130}$$

Here A_0 is the mole fraction of DMT at time t=0. The inverse Laplace transform of Eqn. S130 gives Eqn. S131.

$$A(t) = A_0 \exp\left(-\,(k_1 F + k_3 EF)t\right) \tag{S131}$$

Eqn. S131 was transformed into the Laplace domain resulting in Eqn. S132.

$$sB(s) - B_0 = k_1 A(s)F + k_3 A(s)EF - k_2 B(s)F - k_4 B(s)EF \tag{S132}$$

Here B0 is the initial mole fraction of MCB. The appropriate initial condition is given by S133,

$$B_0 = 0 \tag{S133}$$

and substituting S130 into S132 results in S134.

$$sB(s) = \frac{A_0(k_1 F + k_3 EF)}{s + (k_1 F + k_3 EF)} - k_2 B(s)F - k_4 B(s)EF \tag{S134}$$

Rerrangement of Eqn. S134 in Eqn. S135 results in Eqn. S136.

$$B(s) = \frac{\left(\frac{A_0(k_1 F + k_3 EF)}{s + (k_1 F + k_3 EF)}\right)}{s + (k_2 F + k_4 EF)} \tag{S135}$$

$$B(s) = \frac{A_0(k_1F + k_3EF)}{(s + (k_1F + k_3EF))(s + (k_2F + k_4EF))} \tag{S136}$$

The partial fraction expansion of Eqn. S136 gives Eqn. S137.

$$B(s) = \frac{X}{s} + \frac{Y}{s + (k_1F + k_3EF)} + \frac{Z}{s + (k_2F + k_4EF)} \tag{S137}$$

For simplification, a_1 and a_2 are defined in Eqns. S138-S139.

$$a_1 = (k_1F + k_3EF) \tag{S138}$$

$$a_2 = (k_2F + k_4EF) \tag{S139}$$

Substituting a_1 and a_2 and setting X to 0 gives Eqn. S140.

$$\frac{A_0(k_1F + k_3EF)}{(s + (k_1F + k_3EF))(s + (k_2F + k_4EF))} = \frac{Y}{s + a_1} + \frac{Z}{s + a_2} \tag{S140}$$

Determining the common denominator, and multiplying it to both sides of Eqn. S140 gives Eqn. S141.

$$A_0(k_1F + k_3EF) = Y(s + a_2) + Z(s + a_1) \tag{S141}$$

Combining like terms results in Eqn. S142.

$$A_0(k_1F + k_3EF) = s(Y + Z) + (Ya_2 + Za_1) \tag{S142}$$

Separating into an s term and a constant term yields Eqns. S143-S144

s term	$Y + Z = 0$	(S143)
Constant term	$Ya_2 + Za_1 = A_0(k_1F + k_3EF)$	(S144)

Solving for Z in Eqn. S143 yields Eqn. S145

$$Z = -Y \tag{S145}$$

Substituting S145 into S144 gives S146.

$$A_0(k_1F + k_3EF) = Ya_2 + (-Y)a_1 \tag{S146}$$

Solving for Y results in Eqn. S147.

$$\frac{(A_0(k_1F + k_3EF))}{a_2 - a_1} = Y \tag{S147}$$

Plugging Eqns. 138-139 into Eqn. S147 results in Eqn. S148.

$$Y = \frac{A_0(k_1 + k_3E)}{(k_2 + k_4E) - (k_1 + k_3E)} \tag{S148}$$

Substituting Eqn. S148 into Eqn. S145 yields Eqn. S149.

$$Z = -\frac{A_0(k_1 + k_3E)}{(k_2F + k_4E) - (k_1F + k_3E)} \tag{S149}$$

The inverse Laplace transform of Eqn. S137 results in Eqn. 150.

$$B(t) = X + Yexp(-(k_1F + k_3EF)t) + Zexp(-(k_2F + k_4EF)) \tag{S150}$$

Plugging the values obtained for X, Y, and Z into Eqn. S150 and simplifying, yields the solution in Eqn. S151.

$$B(t) = \frac{A_0(k_1 + k_3E)}{(k_2 + k_4E) - (k_1 + k_3E)} exp(-(k_1F + k_3EF)t) - \frac{A_0(k_1 + k_3E)}{(k_2 + k_4E) - (k_1 + k_3E)} exp(-(k_2F + k_4EF)t) \tag{S151}$$

Eqn. S125 was transformed into the Laplace domain resulting in Eqn. S152.

$$sC(s) - C_0 = k_2B(s)F + k_4B(s)EF \tag{S152}$$

Where C_0 is the initial mole fraction of TPD. An appropriate initial mole fraction of 0 was chosen,

$$C_0 = 0 \tag{S153}$$

yielding Eqn. S154.

$$C(s) = \frac{k_2B(s)F + k_4B(s)EF}{s} \tag{S154}$$

Substituting Eqn. S136 into Eqn. S154 results in Eqn. S155.

$$C(s) = \frac{\left(\frac{A_0(k_1F + k_3EF)}{(s + (k_1F + k_3EF))(s + (k_2F + k_4EF))}\right)(k_2F + k_4EF)}{s} \tag{S155}$$

Simplifying gives Eqn. S156.

$$C(s) = \frac{A_0(k_1F + k_3EF)(k_2F + k_4EF)}{s(s + (k_1F + k_3EF))(s + (k_2F + k_4EF))} \tag{S156}$$

Preforming the partial fraction expansion results in Eqn. S157.

$$C(s) = \frac{X}{s} + \frac{Y}{s + (k_1F + k_3EF)} + \frac{Z}{s + (k_2F + k_4EF)} \tag{S157}$$

For simplification, a_1 and a_2 are defined in Eqns. S158-S159.

$$a_1 = (k_1F + k_3EF) \tag{S158}$$

$$a_2 = (k_2F + k_4EF) \tag{S159}$$

Substituting a_1 and a_2 and setting X to 0 gives Eqn. S160.

$$\frac{A_0(k_1F + k_3EF)(k_2F + k_4EF)}{s(s + a_1)(s + a_2)} = \frac{X}{s} + \frac{Y}{s + a_1} + \frac{Z}{s + a_2} \tag{S160}$$

Determining the common denominator, and multiplying it to both sides of Eqn. S160 gives Eqn. S161.

$$k_1k_2A_0(k_1F + k_3EF) = X(s + a_1)(s + a_2) + Y(s + a_2)s + Z(s + a_1)s \tag{S161}$$

Distributing results in Eqn. S162.

$$A_0(k_1F + k_3EF)(k_2F + k_4EF) = Xs^2 + Xsa_1 + Xsa_2 + Xa_1a_2 + Ys^2 + Ysa_2 + Zs^2 + Zsa_1 \tag{S162}$$

Factoring out like terms with respect to s, results in Eqn. S163.

$$A_0(k_1F + k_3EF)(k_2F + k_4EF) = s^2(X + Y + Z) + s(X(a_1 + a_2) + Ya_2 + Za_1) + Xa_1a_2 \tag{S163}$$

Separating into s^2, s, and constant terms gives Eqns. S164-S166

s^2 term $$X + Y + Z = 0 \tag{S164}$$

s term $$X(a_1 + a_2) + Ya_2 + Za_1 = 0 \tag{S165}$$

Constant term $$A_0(k_1F + k_3EF)(k_2F + k_4EF) = Xa_1a_2 \tag{S166}$$

Substituting Eqns. S158-S159 into Eqn. S166 and simplifying yields Eqn. S167.

$$X = \frac{k_1k_2A_0(k_2F + k_4EF)}{a_1a_2} = \frac{A_0(k_1F + k_3EF)(k_2F + k_4EF)}{(k_1F + k_3EF)(k_2F + k_4EF)} = A_0 \tag{S167}$$

Substituting Eqn. S167 into Eqn. S164 gives Eqn. S168.

$$A_0 + Y + Z = 0 \tag{S168}$$

Solving for Z yields Eqn. S169.

$$Z = -A_0 - Y \tag{S169}$$

Substituting Eqns. S167 and S169 into Eqn. S165, results in Eqn. S170.

$$0 = A_0(a_1 + a_2) + Ya_2 + (-A_0 - Y)a_1 \tag{S170}$$

Rearrangement results in Eqns. S171-S172.

$$-A_0(a_1 + a_2) + A_0a_1 = Ya_2 - Ya_1 \tag{S171}$$

$$\frac{(-A_0a_2)}{a_2 - a_1} = Y \tag{S172}$$

Plugging Eqns. S158-S159 into Eqn. S172 yields Eqn. S173.

$$Y = \frac{-A_0(k_2F + k_4EF)}{(k_2F + k_4EF) - (k_1F - k_3EF)} \tag{S173}$$

Simplification results in Eqn. S174.

$$Y = \frac{-A_0(k_2 + k_4E)}{(k_2 + k_4E) - (k_1 - k_3E)} \tag{S174}$$

Substituting Eqn. S174 into S169 gives S175.

$$Z = \frac{A_0(k_2 + k_4E)}{(k_2 + k_4E) - (k_1 - k_3E)} - A_0 \tag{S175}$$

Rearrangement of Eqn. S175 in Eqns. S176-S177 results in Eqn. S178.

$$Z = A_0\left(\frac{(k_2 + k_4E)}{(k_2 + k_4E) - (k_1 - k_3E)} - 1\right) \tag{S176}$$

$$Z = A_0\left(\frac{(k_2 + k_4E)}{(k_2 + k_4E) - (k_1 - k_3E)} - \frac{(k_2 + k_4E) - (k_1 - k_3E)}{(k_2 + k_4E) - (k_1 - k_3E)}\right) \tag{S177}$$

$$Z = A_0\left(\frac{(k_1 - k_3E)}{(k_2 + k_4E) - (k_1 - k_3E)}\right) \tag{S178}$$

The inverse Laplace transform of Eqn. S157 yields Eqn. S179.

$$C(t) = X + Yexp(-(k_1F + k_3EF)t) + Zexp(-(k_2F + k_4EF)t) \tag{S179}$$

Substituting Eqns. S167, S174, and S178 into Eqn. S179 gives Eqn. S180.

$$\begin{aligned} C(t) = A_0 + \frac{-A_0(k_2 + k_4E)}{(k_2 + k_4E) - (k_1 - k_3E)} exp(-(k_1F + k_3EF)t) \\ + A_0\left(\frac{(k_1 - k_3E)}{(k_2 + k_4E) - (k_1 - k_3E)}\right) exp(-(k_2F + k_4EF)t) \end{aligned} \tag{S180}$$

5.0 5.0 Supplemental figures

5.1 Model fit figures

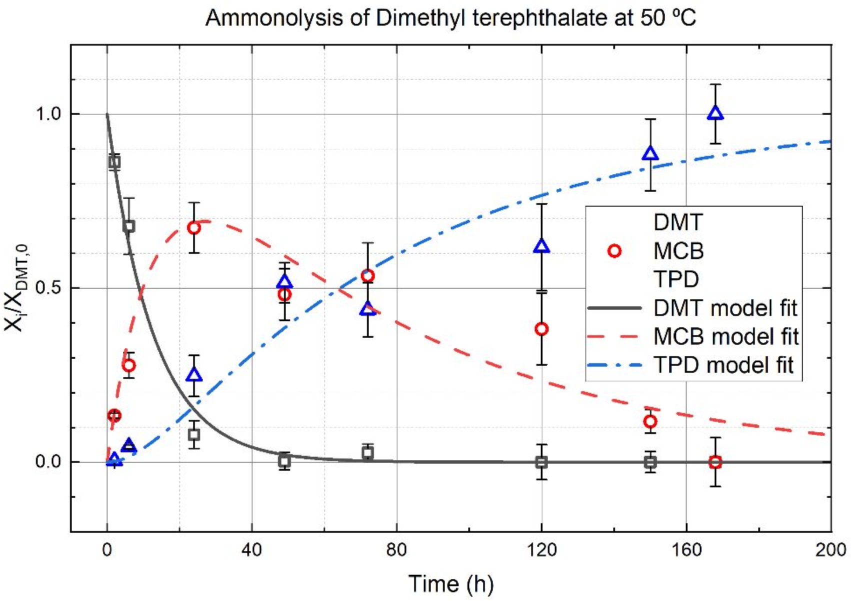

Figure S6. The ammonolysis of DMT with a set point of 50°C. The mole fraction data is plotted as symbols and the lines represent the model fit. The reaction reached completion after 7 days. The k values determined by this fit are .34 h^{-1} and .054 h^{-1} for reaction steps 1 and 2 respectively. The R^2 values are 0.996, 0.890, and 0.926 for DMT, MCB and TPD curves respectively.

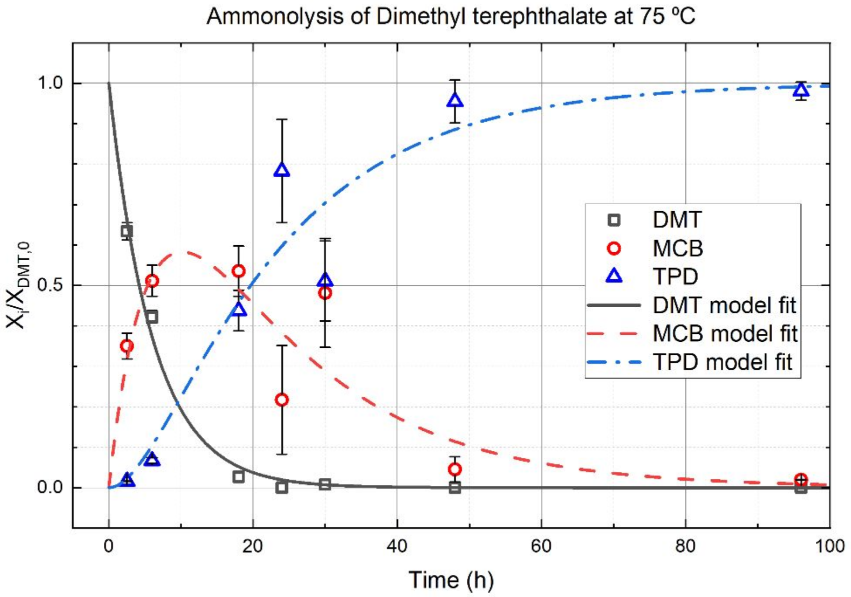

Figure S7. The ammonolysis of DMT at a temperature setpoint of 75°C. The reaction reaches completion at 96h. The mole fraction data is plotted as symbols and the lines represent the model fit. The k values determined by this fit are

.66 h^{-1} and .21h^{-1} for reaction steps 1 and 2 respectively. The R^2 values are 0.995, 0.800, and 0.933 for DMT, MCB and TPD curves respectively.

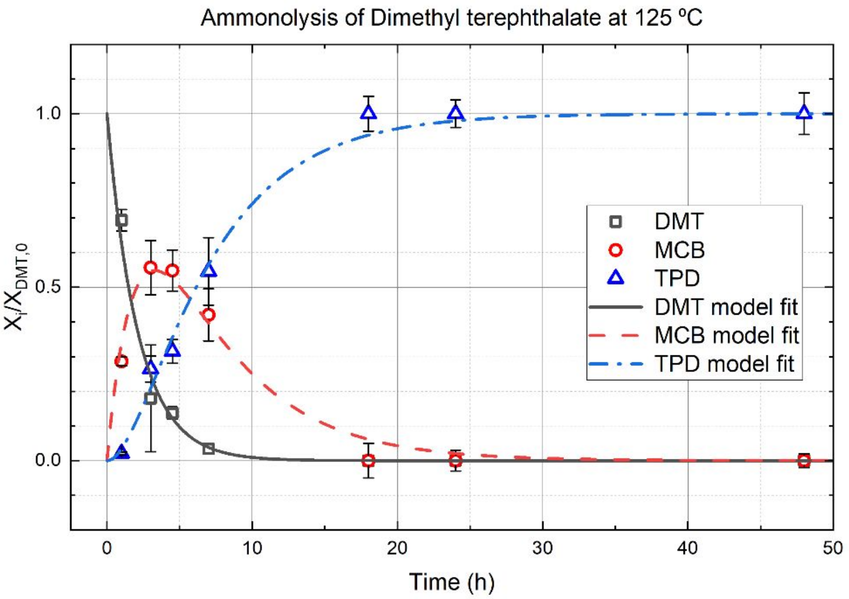

Figure S8. The ammonolysis of DMT at a temperature setpoint of 125^{0}C. The reaction reaches completion at 96h. The mole fraction data is plotted as symbols and the lines represent the model fit. The k values determined by this fit are 1.86 h^{-1} and .73 h^{-1} for reaction steps 1 and 2 respectively. The R^2 values are 0.993, 0.984, and 0.9992 for DMT, MCB and TPD curves respectively.

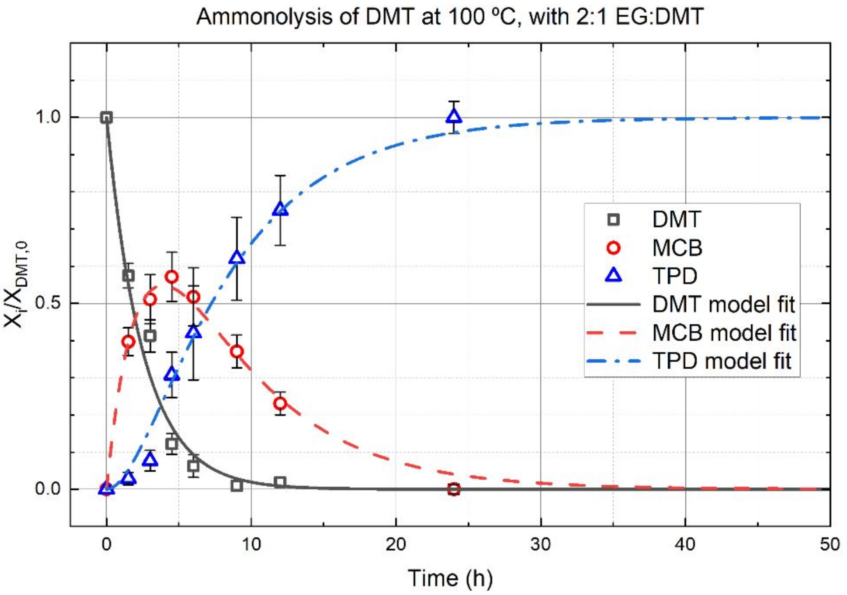

Figure S9. The ammonolysis of DMT at a temperature setpoint of 100^{0}C with a 2:1 EG to DMT ratio. The reaction reaches completion at 30h. The mole fraction data is plotted as symbols and the lines represent the model fit. The R^2 values are 0.986, 0.994, and 0.993 for DMT, MCB and TPD curves respectively.

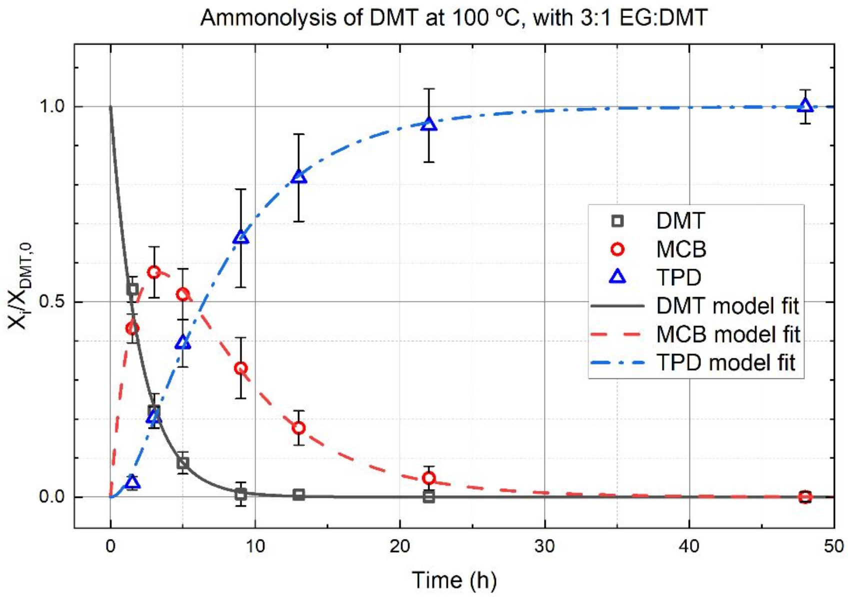

Figure S10. The ammonolysis of DMT at a temperature setpoint of 100°C with a 3:1 EG to DMT ratio. The reaction reaches completion at 30h. The mole fraction data is plotted as symbols and the lines represent the model fit. The R^2 values are 0.998, 0.999, and 0.999 for DMT, MCB and TPD curves respectively.

5.2 Temperature ramp figures

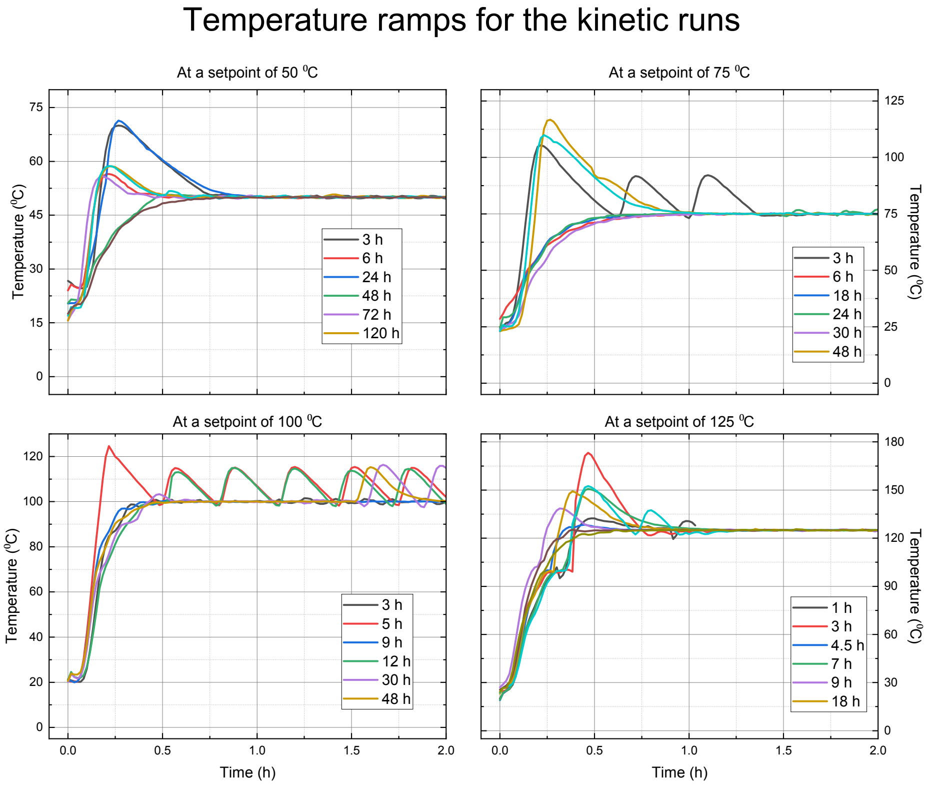

Figure S11. The temperature ramp for each timed run for the ammonolysis of DMT at setpoints of 50°C (top left), 75°C (top right) 100°C (bottom left), 125°C (bottom right). The legend refers to the reaction time. There are some with little to no overshoot and some with large overshoot. These overshoots increase the error of the mole fraction data. To mitigate the error, an average temperature was calculated for each run. The procedure for this calculation is further explained in this document.

5.3 NMR spectra

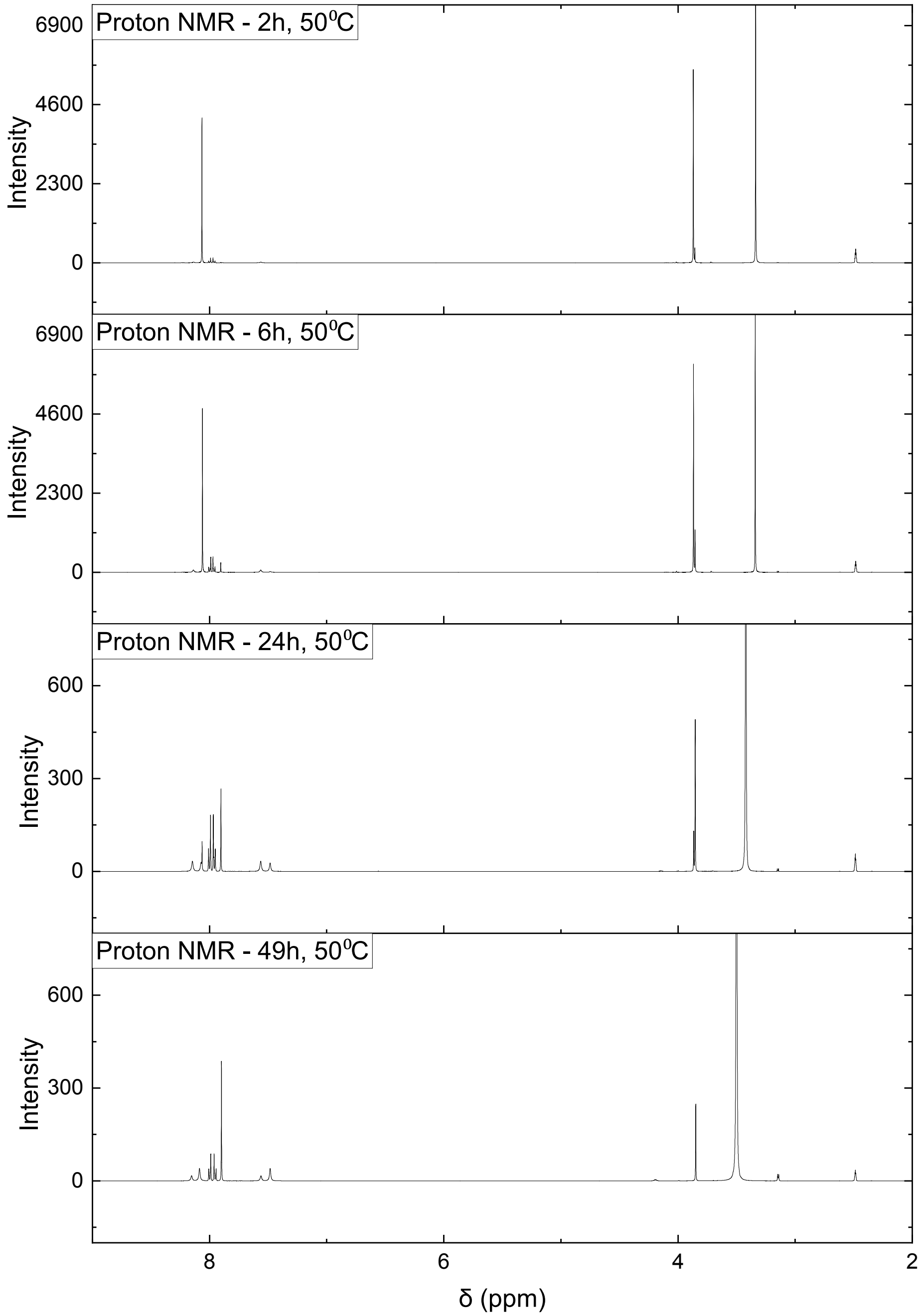

Figure S12. The Proton NMR spectra for the samples taken after 2h, 6h,24h, and 49h of reaction at 50°C. The NMR was taken in DMSO-d6 on a 500 MHz NMR. The relaxation delay was set to 2 seconds and 32 scans were taken. The results of the NMR are as follows. Terephthalamide ^{1}H NMR ((500MHz, DMSO) δ 8.06 (s, 2H), 7.91 (s, 4H), 7.49 (s, 2H)), Dimethyl Terephthalate ^{1}H NMR ((500 MHz, DMSO) δ 8.06 (s, 4H), 3.87 (s, 6H)), and Methyl 4-carbamoylbenzoate (6757-31-9) ^{1}H NMR ((500 MHz, DMSO) δ 8.15 (s, 1H), 8.03 – 7.93 (m, 4H), 7.56 (s, 1H), 3.85 (s, 3H))

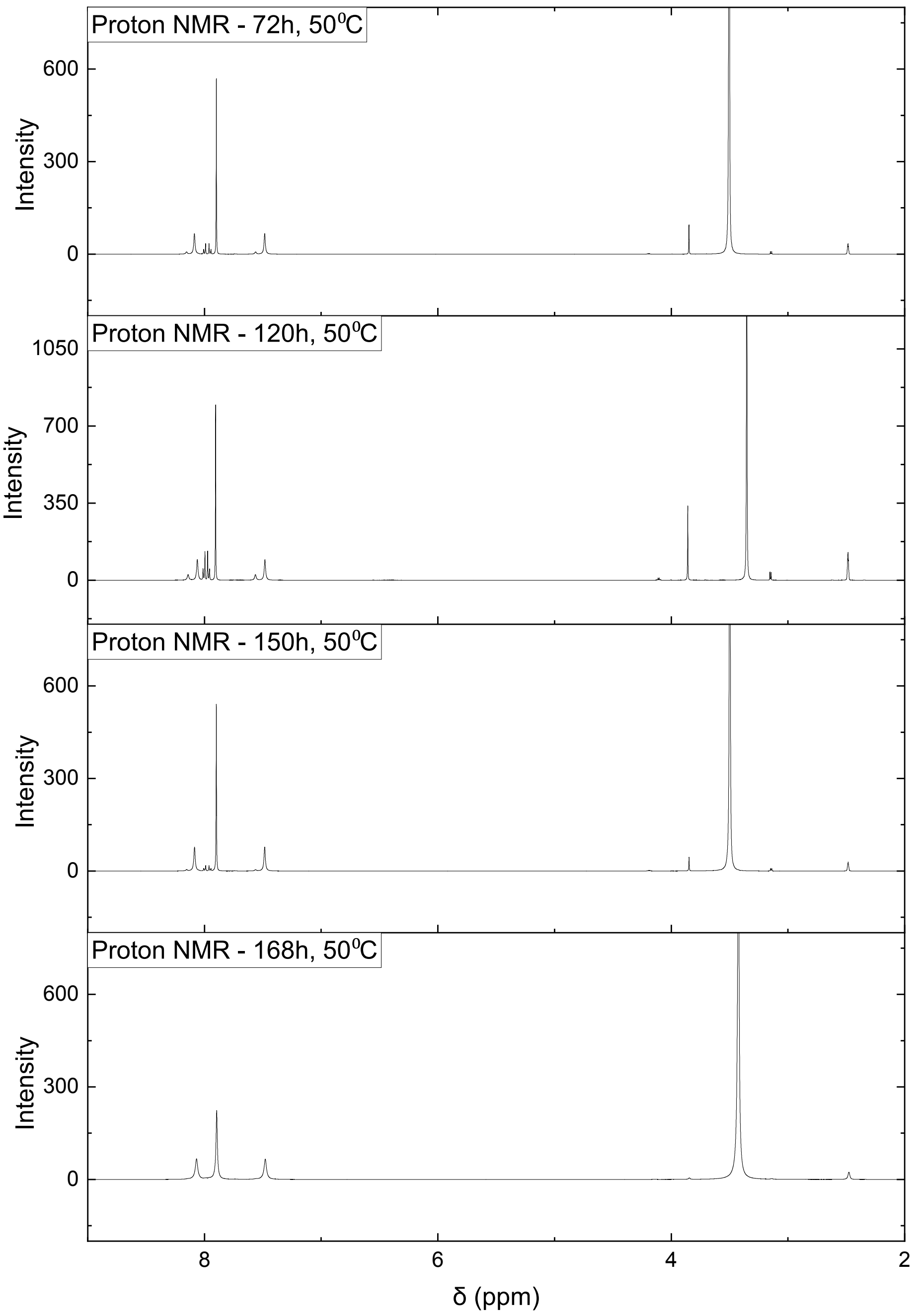

Figure S13. The Proton NMR spectra for the samples taken after 72h, 120h,150h, and 168h of reaction at 50°C. The NMR was taken in DMSO-d6 on a 500 MHz NMR. The relaxation delay was set to 2 seconds and 32 scans were taken. The results of the NMR are as follows. Terephthalamide ^{1}H NMR ((500MHz, DMSO) δ 8.06 (s, 2H), 7.91 (s, 4H), 7.49 (s, 2H)), Dimethyl Terephthalate ^{1}H NMR ((500 MHz, DMSO) δ 8.06 (s, 4H), 3.87 (s, 6H)), and Methyl 4-carbamoylbenzoate (6757-31-9) ^{1}H NMR ((500 MHz, DMSO) δ 8.15 (s, 1H), 8.03 – 7.93 (m, 4H), 7.56 (s, 1H), 3.85 (s, 3H))

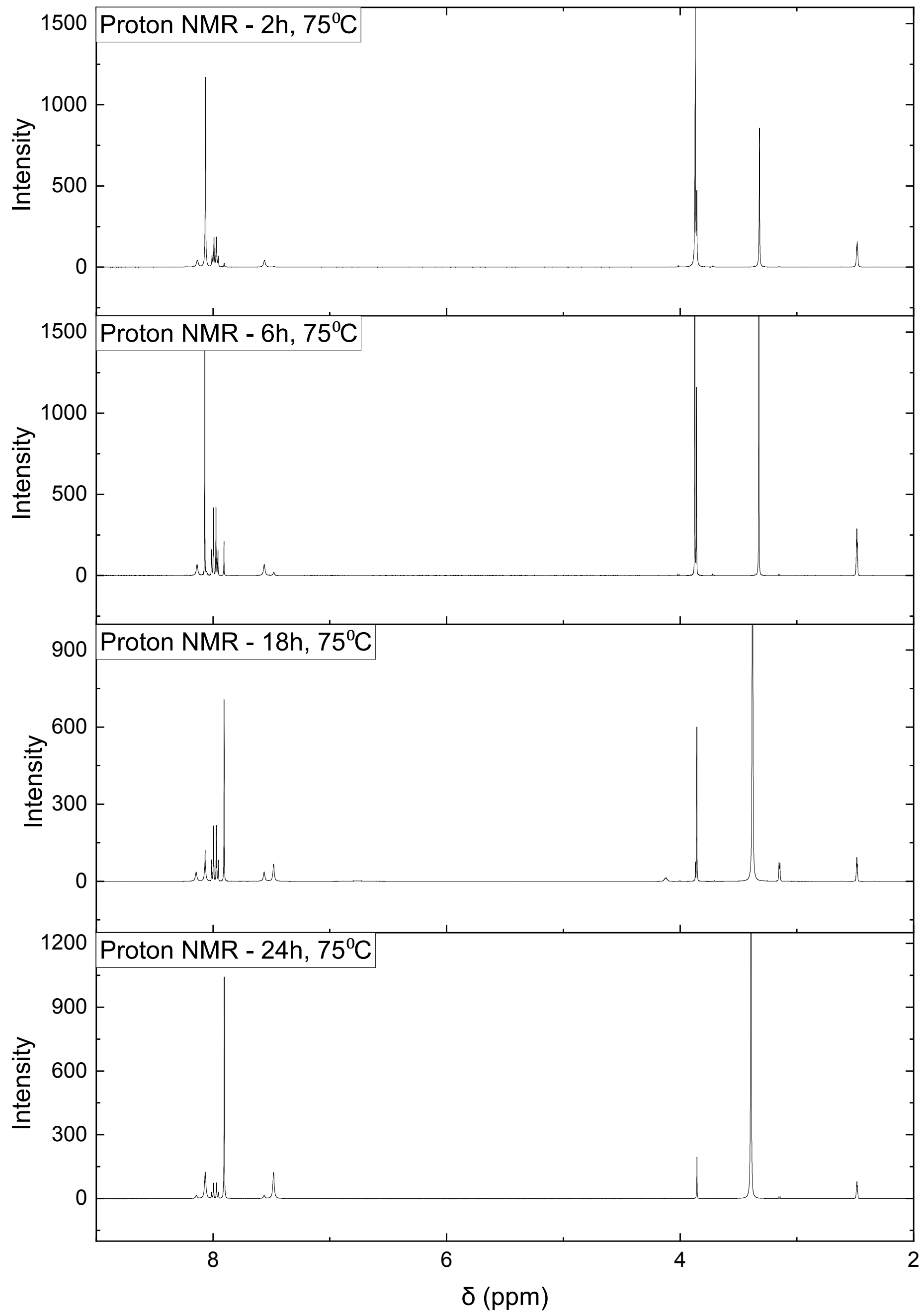

Figure S14. The Proton NMR spectra for the samples taken after 2h, 6h, 18h, and 24h of reaction at 75°C. The NMR was taken in DMSO-d6 on a 500 MHz NMR. The relaxation delay was set to 2 seconds and 32 scans were taken. The results of the NMR are as follows. Terephthalamide ^{1}H NMR ((500MHz, DMSO) δ 8.06 (s, 2H), 7.91 (s, 4H), 7.49 (s, 2H)), Dimethyl Terephthalate ^{1}H NMR ((500 MHz, DMSO) δ 8.06 (s, 4H), 3.87 (s, 6H)), and Methyl 4-carbamoylbenzoate (6757-31-9) ^{1}H NMR ((500 MHz, DMSO) δ 8.15 (s, 1H), 8.03 – 7.93 (m, 4H), 7.56 (s, 1H), 3.85 (s, 3H))

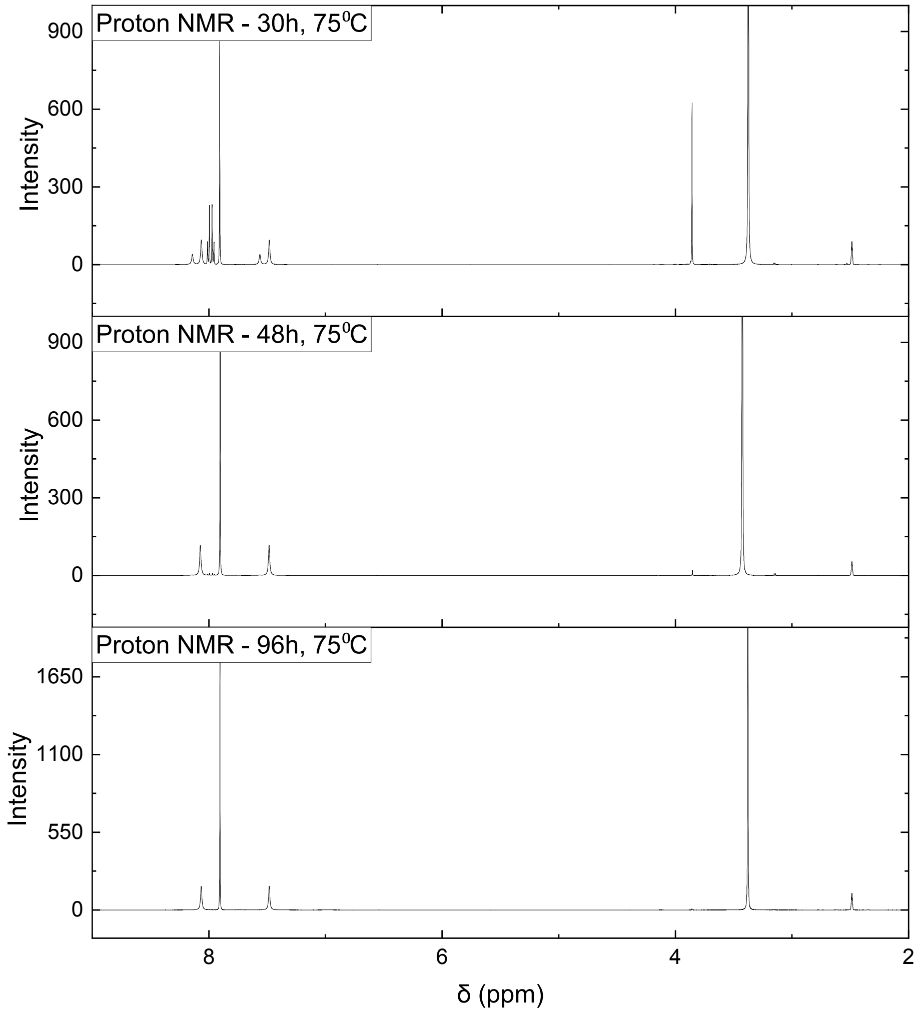

Figure S15. The Proton NMR spectra for the samples taken after 30h, 48h, and 96h of reaction at 75°C. The NMR was taken in DMSO-d6 on a 500 MHz NMR. The relaxation delay was set to 2 seconds and 32 scans were taken. The results of the NMR are as follows. Terephthalamide ^{1}H NMR ((500MHz, DMSO) δ 8.06 (s, 2H), 7.91 (s, 4H), 7.49 (s, 2H)), Dimethyl Terephthalate ^{1}H NMR ((500 MHz, DMSO) δ 8.06 (s, 4H), 3.87 (s, 6H)), and Methyl 4-carbamoylbenzoate (6757-31-9) ^{1}H NMR ((500 MHz, DMSO) δ 8.15 (s, 1H), 8.03 – 7.93 (m, 4H), 7.56 (s, 1H), 3.85 (s, 3H))

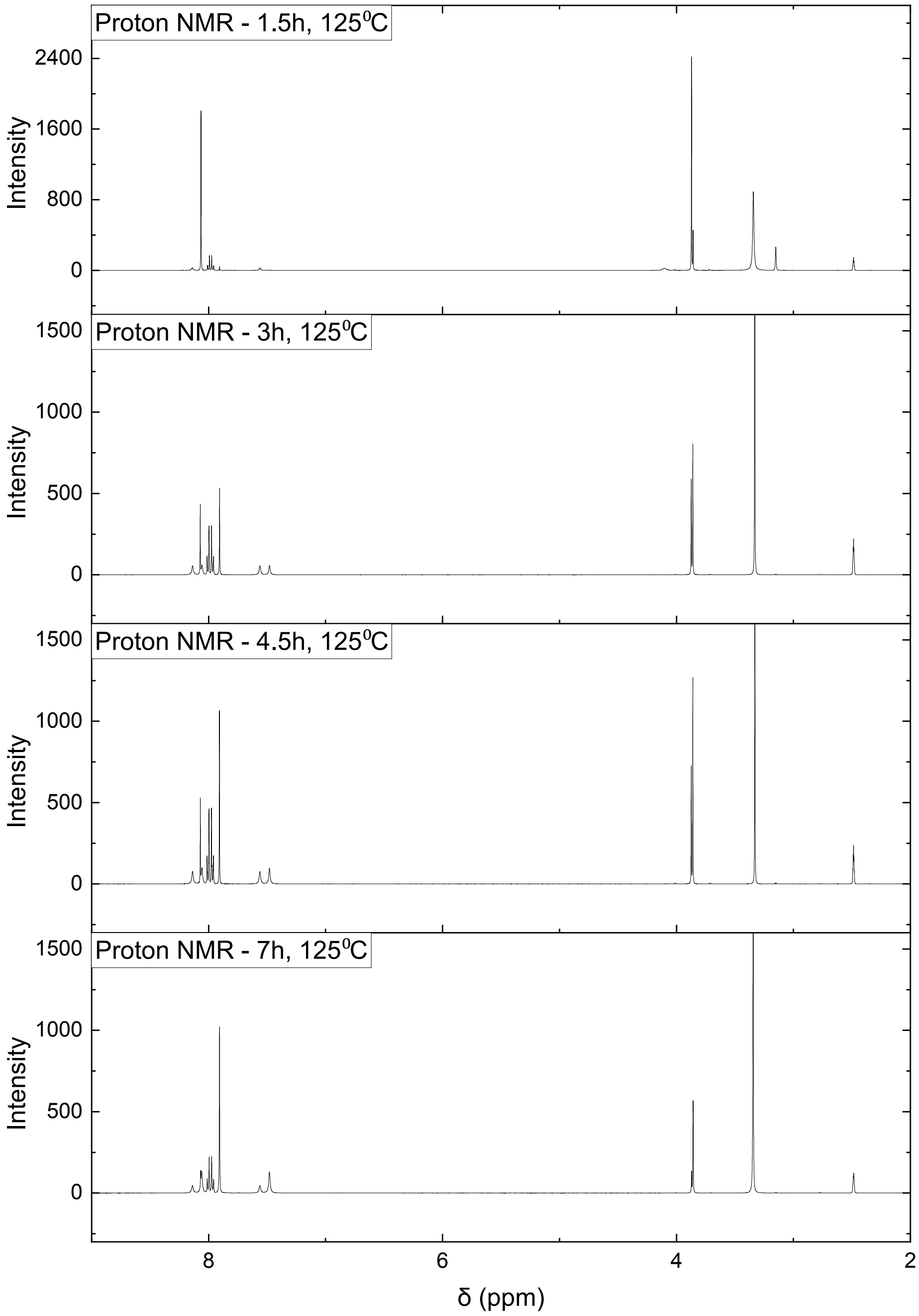

Figure S16. The Proton NMR spectra for the samples taken after 1.5h, 3h, 4.5h, and 7h of reaction at 125°C. The NMR was taken in DMSO-d6 on a 500 MHz NMR. The relaxation delay was set to 2 seconds and 32 scans were taken. The results of the NMR are as follows. Terephthalamide ^{1}H NMR ((500MHz, DMSO) δ 8.06 (s, 2H), 7.91 (s, 4H), 7.49 (s, 2H)), Dimethyl Terephthalate ^{1}H NMR ((500 MHz, DMSO) δ 8.06 (s, 4H), 3.87 (s, 6H)), and Methyl 4-carbamoylbenzoate (6757-31-9) ^{1}H NMR ((500 MHz, DMSO) δ 8.15 (s, 1H), 8.03 – 7.93 (m, 4H), 7.56 (s, 1H), 3.85 (s, 3H))

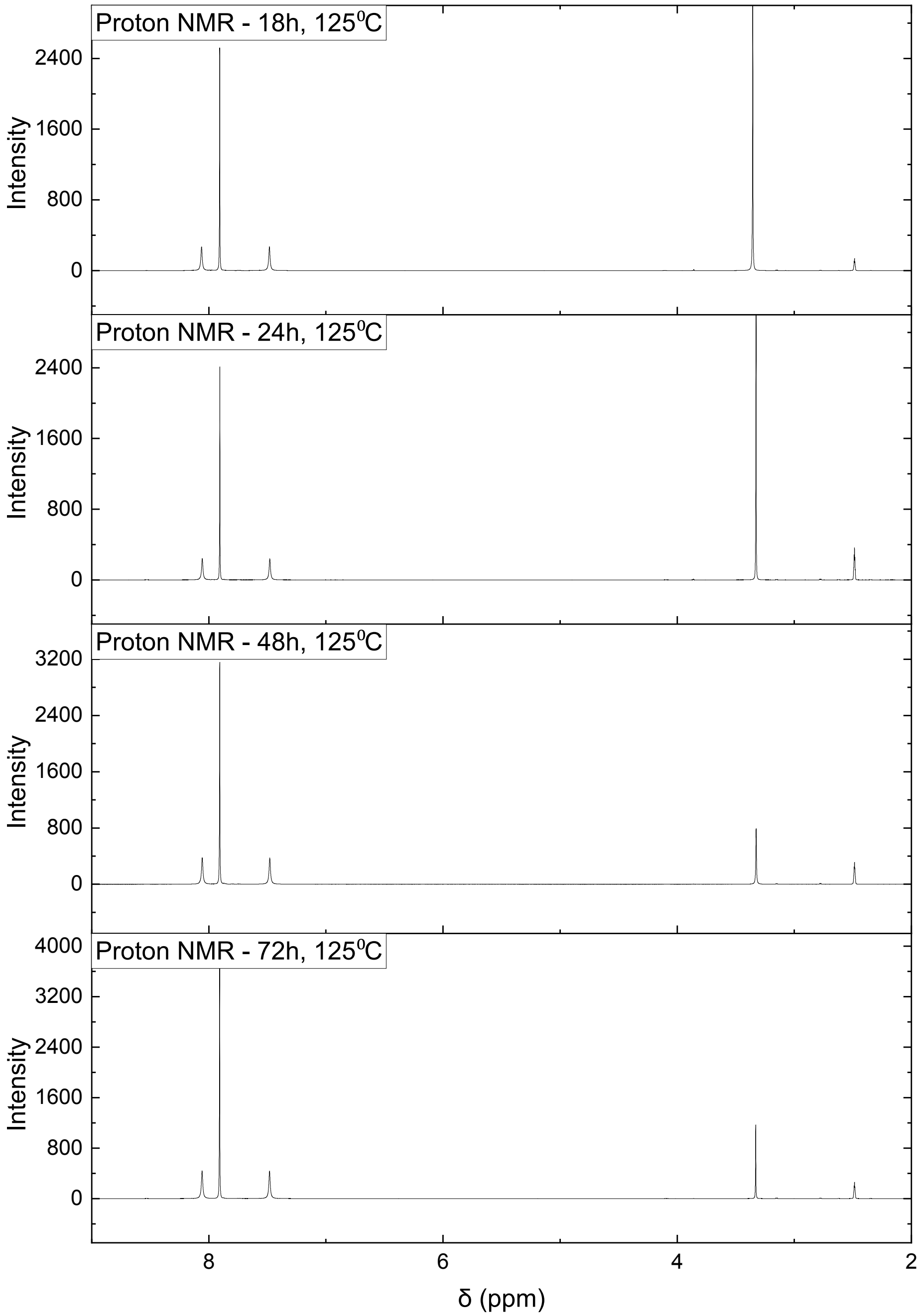

Figure S17. The Proton NMR spectra for the samples taken after 1.5h, 3h, 4.5h, and 7h of reaction at 125°C. The NMR was taken in DMSO-d6 on a 500 MHz NMR. The relaxation delay was set to 2 seconds and 32 scans were taken. The results of the NMR are as follows. Terephthalamide ^{1}H NMR ((500MHz, DMSO) δ 8.06 (s, 2H), 7.91 (s, 4H), 7.49 (s, 2H)), Dimethyl Terephthalate ^{1}H NMR ((500 MHz, DMSO) δ 8.06 (s, 4H), 3.87 (s, 6H)), and Methyl 4-carbamoylbenzoate (6757-31-9) ^{1}H NMR ((500 MHz, DMSO) δ 8.15 (s, 1H), 8.03 – 7.93 (m, 4H), 7.56 (s, 1H), 3.85 (s, 3H))

5.4 Activity model figure

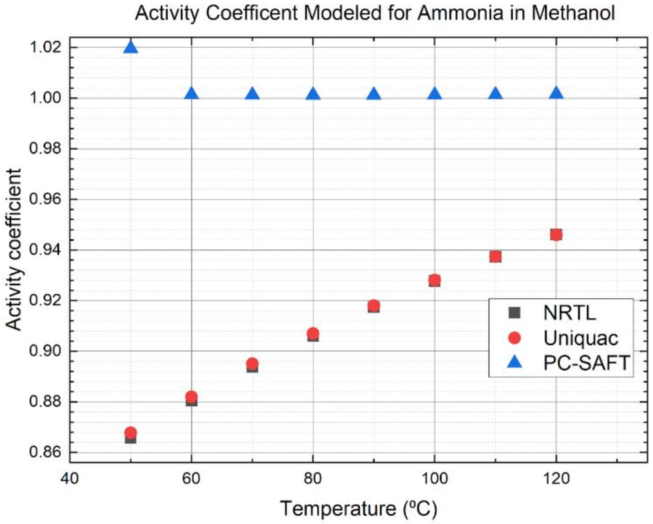

Figure S18. The activity coefficient for ammonia in methanol at 25 mol% ammonia concentration. These values were estimated using ASPEN Plus. The NRTL and Uniquac predictions greatly overlap while the PC-SAFT value is near 1.0 for all temperatures. The PC-SAFT model greatly depends on the available information provided to ASPEN and no such information was found and fed to the model. The NRTL and Uniquac model values are viewed as the most-likely value for the activity coefficients for the solution used in the experiments.

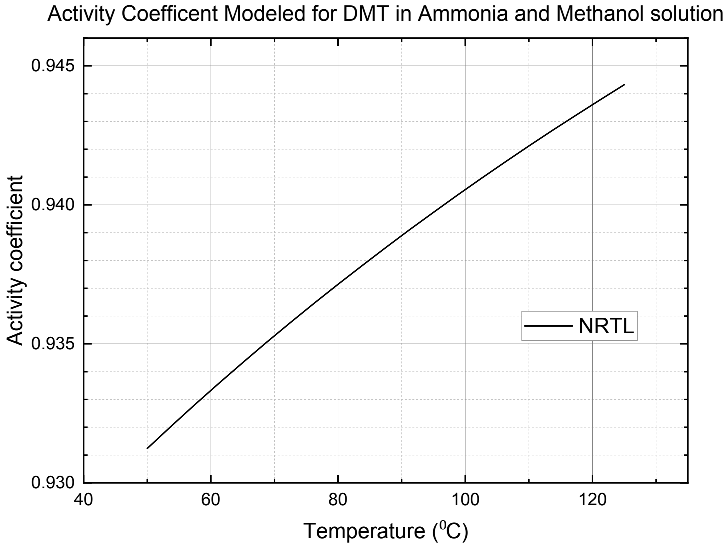

Figure S19. The activity coefficient for 1 mol% DMT in the ammonia in methanol used in this work. These values were estimated using ASPEN Plus and solubility data. The activity coefficient stays near union and shows little change over the defined reaction temperatures.

6.0 6.0 Python Code to determine activation energy

```
import numpy as np
import matplotlib.pyplot as plt
from scipy.optimize import curve_fit,least_squares

# the kinetic model calculated analytically using Laplace transforms
def model2(X, A2, Ea2):
   t,T=X
   DMT0=.012290205
   A0=.250597
   R=8.314
   k1=popt[0]*A0*np.exp(-popt[1]/R/T)
   k2=A2*A0*np.exp(-Ea2/R/T)
   return DMT0*(1+(k2/(k1-k2))*np.exp(-k1*t)-(k1/(k1-k2))*np.exp(-k2*t))
def model1(X, A1, Ea1):
   t,T=X
   DMT0=.012290205
   A0=.250597 #ammonia mole frac
   R=8.314
   k1=A1*A0*np.exp(-Ea1/R/T)
   return -k1*t

#Reorganize the data so that each index represents 1 data point
# for example index 0 is time 2 hrs 51.04C dme=.010595 and tpa=.00005298
#in this code dme corresponds to dimethyl terephthalate and tpa corresponds to terephthalamide
time=np.array([2,6,24,49,72,120,150,168,2.5,6,18,24,30,48,96,3,5,9,13,22,48,100,1.5,3,4.5,7,18,24,48])
Tavg=np.array([51.04,49.62,50.09,49.86,50.29,49.98,50.02,49.96,77.45,73.06,74.40,74.58,74.56,75.16,7
5.04,94.69,100.86,98.41,99.32,99.97,99.64,100.00,105.09,120.94,121.19,122.63,124.31,124.33,124.63])
Tavg=Tavg+273.15
y=np.array([time, Tavg])
#take away the extra zeros for dme and extra 1s for tpa
timedme=np.array([2,6,24,49,72,2.5,6,18,30,3,5,9,13,1.5,3,4.5,7])
Tavgdme=np.array([51.04,49.62,50.09,49.86,50.29,77.45,73.06,74.40,74.56,94.69,100.86,98.41,99.32,1
05.09,120.94,121.19,122.63])
Tavgdme=Tavgdme+273.15
dme=np.array([.010595,.008332,.000972457,.0000316758,.000329055,.007790938,.005186404,.000329
055,.0000941778,.006725146,.003579137,.000700962,.000180738,.008516896,.002205058,.00168359,.
000419175])
ydme=np.array([timedme, Tavgdme])
A0=.250597
logdme=np.log(dme/.012290205)

guess=np.array([4000,25000])
popt,pcov=curve_fit(model1,ydme,logdme,p0=guess)
A1=popt
print(popt)
```

```
T=np.linspace(273,400)
#plt.plot(T,popt[0]*np.exp(-popt[1]/8.314/T))
#k2 is the rate constant for the first reaction at 100C
#idk what k1 is, k3 and k4 are a beginning to look in to error analysis
k2=popt[0]*np.exp(-popt[1]/8.314/373.15)*A0
k1=.96*A0
print(popt[0]*np.exp(-popt[1]/8.314/373.15))

popt2,pcov2=curve_fit(model2,y,tpa,p0=guess)
A2=popt2
print(popt2)
print(popt2[0]*np.exp(-popt2[1]/8.314/373.15))
plt.figure(2)
DMT0=.012290205
t=np.linspace(0,100)
k3=2911*np.exp(-26800/8.314/373.15)*A0
er=.085
k4=((1-er)*4267)*np.exp(-(1+er)*25590/8.314/373.15)*A0
#plt.plot(t,DMT0*np.exp(-k1*t))
#plt.plot(t,DMT0*np.exp(-k2*t))
plt.plot(t,DMT0*np.exp(-k2*t))
plt.plot(t,DMT0*np.exp(-k4*t))
plt.plot(T100time,T100dmt, 'o')

#the error propagated through each data point
error_dme=np.array([.023187,.080839,.009333,.00012,0,0,0,0,.021498,.015218,.006896,0,0,0,0,.02003
7,.03106,.015378,.017625,0,0,0,.009622,.015057,.009245,.012055,0,0,0])
error_tpa=np.array([0,.005779948,.019180299,.057418429,.07781241,.085124219,.103276822,.05,.000
419,.006966,.050634,.087477,.065873,.025279,.022523,.004101,.01515,.048325,.113841,.099125,.0814
23,.030457,.003781,.037261,.03414,.097381,.05,.04,.06])

#with the zeros taken out
error_dme=np.array([.023187,.080839,.009333,.00012,0,.021498,.015218,.006896,0,.020037,.03106,.0
15378,.017625,.009622,.015057,.009245,.012055])
error_tpa=np.array([0,.005779948,.019180299,.057418429,.07781241,.085124219,.103276822,.05,.000
419,.006966,.050634,.087477,.065873,.025279,.022523,.004101,.01515,.048325,.113841,.099125,.0814
23,.030457,.003781,.037261,.03414,.097381,.05,.04,.06])

error_dme=error_dme+.0126 #highest propagated error from measurements
error_tpa=error_tpa +.0126

logerror=np.log((dme+error_dme*dme)/.012290205)

dme_upper=dme+error_dme*dme
tpa_upper=tpa+error_tpa*tpa

dme_lower=dme-error_dme*dme
tpa_lower=tpa-error_tpa*tpa
```

```
popt,pcov=curve_fit(model1,ydme,logerror,p0=guess)
popt2,pcov2=curve_fit(model2,y,tpa_upper,p0=guess)

err1=popt
err2=popt2

print(err1)
print(err2)

popt,pcov=curve_fit(model1,ydme,dme_lower,p0=guess)
popt2,pcov2=curve_fit(model2,y,tpa_lower,p0=guess)

err1_lower=popt
err2_lower=popt2

print(err1_lower)
print(err2_lower)

#pluss or minus error

print(err1-A1)
print(err2-A2)

print(err1[0]*np.exp(-err1[1]/8.314/373.15))
#print(err2[0]*np.exp(-err2[1]/8.314/373.15))
print(err1_lower[0]*np.exp(-err1_lower[1]/8.314/373.15))
#print(err2_lower[0]*np.exp(-err2_lower[1]/8.314/373.15))
print(err1_lower[0]*np.exp(-err1[1]/8.314/373.15))
print(err1[0]*np.exp(-err1_lower[1]/8.314/373.15))
#print(err2_lower[0]*np.exp(-err2[1]/8.314/373.15))
```

7.0 7.0 References for Supporting Information

1. Constantinou, L.; Gani, R., New group contribution method for estimating properties of pure compounds. *AIChE Journal* **1994,** *40* (10), 1697-1710.
